# Cost-effective interventions for breast cancer, cervical cancer, and colorectal cancer: new results from WHO-CHOICE

**DOI:** 10.1186/s12962-018-0157-0

**Published:** 2018-10-29

**Authors:** Ambinintsoa H. Ralaidovy, Chaitra Gopalappa, André Ilbawi, Carel Pretorius, Jeremy A. Lauer

**Affiliations:** 1 Information, Evidence and Research, World Health Organization, Avenue Appia 20, Geneva, Switzerland; 2 Mechanical and Industrial Engineering, 219 Engineering Laboratory, University of Massachusetts, 160 Governors Drive, Amherst, MA 01003-2210 USA; 3 Management of Noncommunicable Diseases, World Health Organization, Avenue Appia 20, Geneva, Switzerland; 4 Avenir Health, 655 Winding Brook Dr 4th Floor, Glastonbury, CT 06033 USA; 5 Health Systems Governance and Financing, World Health Organization, Avenue Appia 20, Geneva, Switzerland

**Keywords:** Cost-effectiveness analysis, Breast cancer, Cervical cancer, Colorectal cancer, Priority setting, Resource allocation, Expansion path, Impact modelling, Intervention costing, Universal health coverage

## Abstract

**Background:**

Following the adoption of the Global Action Plan for the Prevention and Control of NCDs 2013–2020, an update to the Appendix 3 of the action plan was requested by Member States in 2016, endorsed by the Seventieth World Health Assembly in May 2017 and provides a list of recommended NCD interventions. The main contribution of this paper is to present results of analyses identifying how decision makers can achieve maximum health gain using the cancer interventions listed in the Appendix 3. We also present methods used to calculate new WHO-CHOICE cost-effectiveness results for breast cancer, cervical cancer, and colorectal cancer in Southeast Asia and eastern sub-Saharan Africa.

**Methods:**

We used “Generalized Cost-Effectiveness Analysis” for our analysis which uses a hypothetical null reference case, where the impacts of all current interventions are removed, in order to identify the optimal package of interventions. All health system costs, regardless of payer, were included. Health outcomes are reported as the gain in healthy life years due to a specific intervention scenario and were estimated using a deterministic state-transition cohort simulation (Markov model).

**Results:**

Vaccination against human papillomavirus (two doses) for 9–13-year-old girls (in eastern sub-Saharan Africa) and HPV vaccination combined with prevention of cervical cancer by screening of women aged 30–49 years through visual inspection with acetic acid linked with timely treatment of pre-cancerous lesions (in Southeast Asia) were found to be the most cost effective interventions. For breast cancer, in both regions the treatment of breast cancer, stages I and II, with surgery ± systemic therapy, at 95% coverage, was found to be the most cost-effective intervention. For colorectal cancer, treatment of colorectal cancer, stages I and II, with surgery ± chemotherapy and radiotherapy, at 95% coverage, was found to be the most cost-effective intervention.

**Conclusion:**

The results demonstrate that cancer prevention and control interventions are cost-effective and can be implemented through a step-wise approach to achieve maximum health benefits. As the global community moves toward universal health coverage, this analysis can support decision makers in identifying a core package of cancer services, ensuring treatment and palliative care for all.

**Electronic supplementary material:**

The online version of this article (10.1186/s12962-018-0157-0) contains supplementary material, which is available to authorized users.

## Background

Although not specifically mentioned in the millennium development goals (MDGs), cancer is now addressed in target 3.4 of the sustainable development goals (SDGs), which aims to reduce premature mortality related to noncommunicable diseases (NCDs). Cancer is one of the main causes of morbidity and mortality worldwide, with the incidence of new cases expected to rise by 70% in the next two decades [1]. Between 2000 and 2015, cancer deaths globally increased from 7 million to 8.8 million deaths each year, accounting for 1 in 6 of all deaths globally and the largest relative increase has been in low- and middle-income countries, where health systems are least prepared to manage the cancer burden [2]. While communicable disease deaths have decreased 26% between 2000 and 2015, deaths from cancer have increased 26%, with a significant increased proportion of cancer-related deaths occurring in Asia and Africa [2, 3]. Cervical cancer and breast cancer are the leading causes of cancer-related death among women in the sub-Saharan Africa region, resulting in, respectively, 23.2% and 19.3% [3] of total cancer deaths; colorectal cancer is one of the most common causes of cancer-related death for both sexes worldwide [3]. The total annual economic costs of cancer globally was estimated at approximately US$ 1.16 trillion in 2010 and has continued to rise, threatening health budgets and economies at all income levels and also causing financial catastrophe for individuals and families [3].

Following the adoption of the Global Action Plan for the Prevention and Control of NCDs 2013–2020 in 2013 [4], an update to Appendix 3 of the action plan was requested by the Member States in 2016 [5]. The update, which provides a list of recommended NCD interventions, was endorsed by the Seventieth World Health Assembly in May 2017. These priority NCD interventions, if implemented to scale, would enable countries to make significant progress to reduce by 25% the number of the NCD-related premature death by 2025 [6].

To achieve these targets and those specified in the United Nations Agenda for Sustainable Development, cancer screening programs need to become more systematic and reach a more significant proportion of their target populations in Southeast Asia and eastern sub-Saharan Africa. Data from the WHO Country Capacity Survey 2015 found that countries in WHO South-East Asia (SEAR) and Africa regions (AFR) were the least likely among WHO Regions to have a breast screening program with 64% and 57% availability respectively. However, the majority of screening programs reached less than 10% coverage in these regions. Human papillomavirus (HPV) vaccination was available in approximately 50% of countries in AFR and almost 20% in SEAR, similarly with the majority reaching less than 10% coverage. Cancer centres or cancer departments were available in approximately 55% of countries in SEAR and 30% in AFR. Treatment, including cancer surgery and subsidized chemotherapy, and palliative care services were also generally unavailable to the majority of countries [7].

The main contribution of this paper is to present results of analyses used to identify how decision makers can achieve maximum health gain using the cancer interventions in Appendix 3 of the global action plan. We also present methods used to calculate new WHO-CHOICE cost-effectiveness results for breast cancer, cervical cancer, and colorectal cancer. The “expansion paths” we present are a proposed sequence in which interventions could be adopted to achieve the maximum health gain. The order in which each intervention or combination of interventions appears on the line is based on the incremental costs and effectiveness of each intervention compared to the last one on the line [8].

## Methods

We used Generalized Cost-Effectiveness Analysis (GCEA) for our analysis, which is an approach recommended by WHO-CHOICE and details of which have been published previously [8–10]. In this paper, we describe the methods related to breast cancer, cervical cancer, and colorectal cancer.

We did not analyze all possible combinations of interventions for these three cancers, an approach which has been previously studied [11]. Instead, we emphasize a package of interventions relevant to a comprehensive cancer control programme. A “comprehensive cancer control approach” consists of prevention, early diagnosis and screening linked to treatment, palliative care, and survivorship care [12]. We focus moreover on those aspects of comprehensive cancer control that are generalizable to all resource settings. Furthermore, based on previous work on cancer [13, 14], the use of an approach based on comprehensive cancer control has been found to be justified on grounds of cost effectiveness.

We considered aspects of the expansion path that take into account specific programmatic concerns. This means that, if a particular technology appears on the expansion path at a certain level of coverage, then for the next step, we considered the most cost effective interventions that included this particular technology at the same or higher coverage, since a decision maker would likely not wish to bring a particular intervention up to scale only to replace it with a competing technology when higher levels of resources are available.

Our analysis is restricted to Southeast Asia and eastern sub-Saharan Africa [15] and uses epidemiological and cost data for a base year of 2010. These two regions were selected as they are geographically and epidemiologically diverse regions which will provide differing examples of cost-effectiveness results and, we predicted, would have different findings. These regions are a WHO-CHOICE level feature across 20 diseases/risk factors. A generic approach is required for standardization. The results are intended to be indicative examples, rather than prescriptive packages for countries to implement. Health outcomes are reported as the gain in healthy life years (HLYs) and are estimated using a dynamic simulation model in the Spectrum software. HLYs are presented both undiscounted and discounted at 3% per annum [8]. Disability weights (DWs) were obtained from the Global Burden of Disease (GBD) study 2010 [16]. All health system costs required to deliver the intervention are included, regardless of payer. Costs include patient-level delivery costs as well as programme-level (i.e. overhead) costs [17]. A 3% per annum discount is applied to costs in all scenarios [8]. Programmes are considered to be implemented for 100 years. Each individual and combined intervention is evaluated at 50%, 80% and 95% coverage levels [17].

### Impact modelling

#### Interventions

This paper analyses 14 individual and combination interventions: 9 for cervical cancer, 3 for breast cancer, and 2 for colorectal cancer.

These interventions are listed in Table 1. All interventions are first compared to the “null,” a hypothetical scenario where the effects of all currently implemented interventions are removed. Following the definition of the null, the marginal effects and costs of each intervention or combination are evaluated.

**Table 1:** Interventions included in the analysis

Disease	Label	Interventions [22]
Cervical cancer	C1a	Vaccination against human papillomavirus (two doses) of 9–13-year-old girls
	C1b	Prevention of cervical cancer by screening women aged 30–49 through visual inspection with acetic acid linked with timely treatment of pre-cancerous lesions
	C1c	Prevention of cervical cancer by screening women aged 30–49 through Pap smear (cervical cytology) every 3–5 years linked with timely treatment of pre-cancerous lesions
	C1d	Prevention of cervical cancer by screening women aged 30–49 through human papillomavirus test every 5 years linked with timely treatment of pre-cancerous lesions
	C1e	Vaccination against human papillomavirus (two doses) of 9–13-year-old girls and prevention of cervical cancer by screening women aged 30–49 through visual inspection with acetic acid linked with timely treatment of pre-cancerous lesions
	C1f	Vaccination against human papillomavirus (two doses) of 9–13-year-old girls and prevention of cervical cancer by screening women aged 30–49 through Pap smear (cervical cytology) every 3–5 years linked with timely treatment of pre-cancerous lesions
	C1g	Vaccination against human papillomavirus (two doses) of 9–13-year-old girls and Prevention of cervical cancer by screening women aged 30–49 through human papillomavirus test every 5 years linked with timely treatment of pre-cancerous lesions
	C1h	Treatment of cervical cancer stages I and II with either surgery or radiotherapy ± chemotherapy
	C1i	Basic palliative care for cancer: home-based and hospital care with multi-disciplinary team and access to opiates and essential supportive medicines
Breast cancer	C2a	Treatment of breast cancer stages I and II with surgery ± systemic therapy
	C2b	Screening with mammography (once every 2 years for women aged 50–69 years) linked with timely diagnosis and treatment of breast cancer
	C2c	Basic palliative care for cancer: home-based and hospital care with multi-disciplinary team and access to opiates and essential supportive medicines
Colorectal cancer	C3a	Treatment of colorectal cancer stages I and II with surgery ± chemotherapy and radiotherapy
	C3b	Basic palliative care for cancer: home-based and hospital care with multi-disciplinary team and access to opiates and essential supportive medicines

Interventions are based on WHO Guidance for cervical cancer [18], for breast cancer [6, 14, 19, 20] and for colorectal cancer [14, 19–21]. These guidelines emphasize comprehensive cancer control including diagnosis, staging, multi-modality treatment, survivorship care and palliative care.

#### Estimation of HLYs

Health outcomes were estimated using a deterministic state-transition cohort simulation (Markov model). In this type of simulation, healthy stages and disease stages, distributed by age, are modelled as the exhaustive and mutually exclusive states of a Markov model, i.e. at any cross-sectional point in time, all persons in the population belong to one and only one of the states. As persons age, they transition between states based on state-specific transition rates. They can either remain in the healthy state, or transition from healthy to the initial disease state, representing disease onset, and then transition between subsequent disease states, representing either progression to an advanced state of disease or regression to a lower disease state or back to the healthy state. Regression to healthy state is modelled only for pre-cancerous states. By representing preclinical and clinical disease stages as separate states, diagnoses are modelled through transitions from preclinical to clinical states. Persons can transition to mortality from any state, at which point they leave the model. To model the impact of disease and treatment, different rates are used for transitioning to mortality, e.g. higher rates are applied to more advanced stages of disease to represent the reduced effectiveness of treatment. A brief outline of the state transitions specific to each type of cancer are discussed below, and detailed flow diagrams are presented in Additional file 1. The model is discussed in more detail in [23].

In the absence of an intervention, transitions are based on natural rates of progression or regression. With an intervention, rates of transitions are modified, e.g. the rates from healthy to HPV state are decreased to represent the effectiveness of vaccination, or the rates from preclinical to clinical states are increased, such that more persons are diagnosed in early stages of disease to represent effective screening. The health outcomes of interventions are measured as a relative increase in healthy life years lived in an intervention scenario compared to no intervention. Healthy life years are calculated as the sum of person-time in all states (except mortality) after discounting for disability specific to each state (see “Disability weights”).

##### Cervical cancer

The vast majority of cervical cancer cases originate as human papillomavirus (HPV) infection, a sexually transmitted disease. Therefore, the cervical cancer state-transition model consisted of three components: HPV transmission, pre-cancerous HPV progression and regression, and cervical cancer progression. HPV subtypes were categorized into three groups: (i) HPV 16/18 (which contributes to an estimated 70% [24] of all cervical cancers), (ii) HPV high-risk (all HPV types other than 16/18 that are at high-risk of progressing to cancer), and (iii) HPV low-risk (all other types that have a low-risk of progressing to cancer). Co-infection with multiple subtypes was not modelled.

It was assumed that women in the healthy state can become infected with one of the three HPV categories through sexual contact with an infected partner. Therefore, the rates of transition from healthy to HPV states were determined dynamically through a transmission model. In the pre-cancerous part of the model, persons in the HPV+ state could progress to cervical intra-epithelial neoplasia (CIN), subsequently to a low-grade dysplasia CIN-1, and then advance to CIN-2-3. Persons in CIN stages could naturally regress in disease stage and have HPV clearance, or could regress to HPV upon screening and treatment. Upon regression, there was short-term immunity to HPV before transitioning to healthy state that re-exposed persons to infection. From CIN-2-3, persons could progress to invasive cancer, first to carcinoma in situ (CIS), and further to states I, II, III, and IV. From any of these states, persons could transition from pre-clinical to clinical states through diagnosis based on symptoms or through screening. In men, we did not model cancers related to HPV, but only modelled HPV infection, transmission and natural regression. Detailed flow diagrams of the state transitions are presented in Additional file 1.

##### Breast cancer

We assumed that breast cancer initiated directly as carcinoma in situ (CIS), i.e., women could transition from healthy to CIS then progress to invasive carcinoma stages I, II, III, and IV. From any of these disease states, persons could transition from pre-clinical to clinical states through diagnosis based on symptoms or through screening.

##### Colorectal cancer

We assumed that about 77% of colorectal cancers originate as pre-cancerous polyps and the remaining 23% originate directly as carcinoma in situ (CIS) [25, 26]. We have divided the pre-cancerous states into three different sizes of polyps (≤ 5 mm, 6–9 mm, ≥ 10 mm) because of the variation in effectiveness of treatment by polyp size [27]. Upon transition to CIS, disease progresses through invasive carcinoma stages I, II, III, and IV. From any of these states, persons can transition from pre-clinical to clinical states through diagnosis based on symptoms or through screening.

#### Data sources for state-transition rates

We assumed that natural rates of transition from healthy to first stage of disease and from preclinical to clinical states, i.e., in the absence of a controlled intervention program, were specific to the population. These population-specific parameters were estimated using a newly developed Markov-process methodology that is described in elsewhere [23] and summarized in Additional file 1. We assumed that rates of natural progression and regression between disease states are specific to the cancer but do not vary by population. We extracted these parameters from the literature (see Additional file 1).

Each major cancer group (i.e., breast, cervical and colorectal) and each stage of disease has unique values for transition parameters to account for variations in the tumor biology and progression of cancer. It is likely that there are also differences in the natural history and tumor biology between the different molecular subtypes within each of these major cancer groups. Currently, the published studies in LMIC from which parameters are generated have not generally distinguished between these molecular subtypes. However, the parameters of the model do allow for greater specificity that can be used as more data on the diagnosis and treatment of cancer subtypes becomes available—for example, the diagnostic rates and impact of trastuzumab for HER2+ breast cancer in LMIC, which does have a distinct natural history and impact of this particular treatment strategy.

#### Intervention effect sizes

##### Disability weights

Disability weights (DWs) for each health state were drawn from the disability weight study of the Global Burden of Disease 2010 [16] and can be found in Additional file 2, Table S2.

GBD provides DWs for the following general cancer stages: “cancer: diagnosis and primary therapy”, “Cancer: metastatic”, “terminal phase: with medication (for cancers, end-stage kidney or liver disease)”, “terminal phase: without medication (for cancers, end-stage kidney or liver disease)”, as well as “mastectomy” and “stoma” cancer-specific stages/states.

For all three cancer types, we obtained DWs for all pre-terminal cancer phases without treatment, by inflating the “cancer: diagnosis and primary therapy” DW estimate by the ratio between the two DW estimates provided for terminal cancer without treatment. For the terminal cancer stage of each cancer type, we used the GBD estimate directly.

For the DWs for cancer with treatment, for the terminal cancer stage of each cancer type, we used the GBD estimate directly. For the pre-terminal cancer stages we followed a disease-specific approach, as described following.

##### Cervical cancer

For the early stages (0–II) we used the DW for “cancer: diagnosis and primary therapy” and applied a correction for the percentage of cases in the first year of treatment only. For stage III we did not apply a correction.

##### Breast cancer

For early stages (I and II) we used a weighted average DW for “cancer: diagnosis and primary therapy” and “mastectomy”, and applied the DW to the percentage of cases in the first year of treatment only. For stage III we used “cancer: diagnosis and primary therapy”, and we applied the DW to the percentage of cases in the first year of treatment in that stage.

##### Colorectal cancer

We used a weighted average DW for “cancer: diagnosis and primary therapy” and “stoma” for the estimated 5% of patients who would require a stoma, and adjusted the part corresponding to “cancer: diagnosis and primary therapy” for the percentage of cases in the first year of treatment in that stage only, except for stage III for which we did not apply this correction.

##### Incidence

For all three cancers, incidence estimates and age at diagnosis are sourced from GLOBOCAN [21].

For cervical cancer, estimates of HPV distribution by type are taken from [28–30]. Transition rates from dysplasia (CIN) to carcinoma are taken from [31].

All effect sizes can be found in the Additional file 2.

### Intervention costing

We followed a standardized framework developed for WHO-CHOICE to cost all the interventions. We used an “ingredients based” approach, whereby each input required for the intervention is identified and valued. We have included costs incurred at the point of delivery such as drugs and supplies, and health facility visits (including health workforce costs), as well as programmatic costs such as administration, monitoring and evaluation, supervision, training [17]. Programmatic costs for cancer screening include administrative costs, quality assurance and monitoring and evaluation, estimated at approximately 20% of total costs [32]. Screening programme costs include follow-up diagnostic tests for false positive screening results. All intervention costs are calculated assuming that the health system capacity is available to support the intervention. Lists of consumables were identified from WHO Priority Medical Devices in Cancer Management 2017 [33]. Consumables required include those needed for treatment-related complications and surveillance after treatment completion. Systemic therapy treatment regimens were taken from WHO List of Essential Medicines [34]. Prices were taken from the MSH drug price database as median buyer price [35] and from the WHO-CHOICE price database [17]. Costs in all scenarios were discounted at 3% per annum. Costs are reported in 2010 International dollars. Costing assumptions can be found in Additional file 2.

## Results

Costs, HLYs gained, and the cost effectiveness associated with each intervention are presented in Tables 2 and Table  3. These tables present only the most cost-effective interventions on the sectoral expansion path for all three cancers. Interventions that are “dominated” i.e. are more costly or less effective, are presented in cancer-specific tables (see Additional file 2).

**Table 2:** Costs, effects and incremental cost-effectiveness of cancer interventions in Southeast Asia

Label	Description of the intervention	Pop° coverage (%)	Costs per 10 million population (million I$ 2010)	HLY per 10 million population (undiscounted)	Average cost-effectiveness ratio (ACER)	Incremental cost-effectiveness ratio (ICER)
CVC_C1e	Vaccination against human papillomavirus (two doses) of 9–13-year-old girls and prevention of cervical cancer by screening women aged 30–49 through visual inspection with acetic acid linked with timely treatment of pre-cancerous lesions	50	396	4,541,842	87	87
CRC_C3a	Treatment of colorectal cancer stages I and II with surgery ± chemotherapy and radiotherapy	95	207	870,417	238	238
BRC_C2a	Treatment of breast cancer stages I and II with surgery ± systemic therapy	95	206	816,200	252	252
CVC_C1e	Vaccination against human papillomavirus (two doses) of 9–13-year-old girls and prevention of cervical cancer by screening women aged 30–49 through visual inspection with acetic acid linked with timely treatment of pre-cancerous lesions	80	549	5,106,391	108	272
CVC_C1e	Vaccination against human papillomavirus (two doses) of 9–13-year-old girls and prevention of cervical cancer by screening women aged 30–49 through visual inspection with acetic acid linked with timely treatment of pre-cancerous lesions	95	626	5,262,580	119	491
BRC_C2b	Screening with mammography (once every 2 years for women aged 50–69 years) linked with timely diagnosis and treatment of breast cancer	95	1056	1,627,782	649	1048
BRC_C2c	Basic palliative care for breast cancer: home-based and hospital care with multi-disciplinary team and access to opiates and essential supportive medicines	95	193	22,877	8434	8434
CRC_C3b	Basic palliative care for colorectal cancer: home-based and hospital care with multi-disciplinary team and access to opiates and essential supportive medicines	95	158	5944	26,571	26,571
CVC_C1i	Basic palliative care for cervical cancer: home-based and hospital care with multi-disciplinary team and access to opiates and essential supportive medicines	95	156	5262	29,704	29,704

*CVC* cervical cancer, *BRC* breast cancer, CRC colorectal cancer

**Table 3:** Costs, effects and incremental cost-effectiveness of cancer interventions in Eastern sub-Saharan Africa

Label	Description of the intervention	Pop° coverage (%)	Costs per 10 million population (I$ 2010)	HLY per 10 million population (undiscounted)	Average cost-effectiveness ratio (ACER)	Incremental cost-effectiveness ratio (ICER)
CVC_C1a	Vaccination against human papillomavirus (two doses) of 9–13-year-old girls	50	146	5,215,136	28	28
CVC_C1a	Vaccination against human papillomavirus (two doses) of 9–13-year-old girls	80	190	6,773,262	28	28
CVC_C1e	Vaccination against human papillomavirus (two doses) of 9–13-year-old girls and prevention of cervical cancer by screening women aged 30–49 through visual inspection with acetic acid linked with timely treatment of pre-cancerous lesions	80	1163	30,421,065	38	41
BRC_C2a	Treatment of breast cancer stages I and II with surgery ± systemic therapy	95	157	1,389,662	113	113
CVC_C1e	Vaccination against human papillomavirus (two doses) of 9–13-year-old girls and prevention of cervical cancer by screening women aged 30–49 through visual inspection with acetic acid linked with timely treatment of pre-cancerous lesions	95	1362	31,554,286	43	175
CRC_C3a	Treatment of colorectal cancer stages I and II with surgery ± chemotherapy and radiotherapy	95	136	626,379	217	217
BRC_C2b	Screening with mammography (once every 2 years for women aged 50–69 years) linked with timely diagnosis and treatment of breast cancer	95	1307	2,697,617	485	485
BRC_C2c	Basic palliative care for breast cancer: home-based and hospital care with multi-disciplinary team and access to opiates and essential supportive medicines	95	171	56,749	3009	3009
CVC_C1i	Basic palliative care for cervical cancer: home-based and hospital care with multi-disciplinary team and access to opiates and essential supportive medicines	95	161	48,488	3316	3316
CRC_C3b	Basic palliative care for colorectal cancer: home-based and hospital care with multi-disciplinary team and access to opiates and essential supportive medicines	95	113	5602	20,117	20,117

*CVC* cervical cancer, *BRC* breast cancer, *CRC* colorectal cancer

For cervical cancer, vaccination against human papillomavirus (two doses) of 9–13-year-old girls combined with prevention of cervical cancer by screening women aged 30–49 through visual inspection with acetic acid linked with timely treatment of pre-cancerous lesions (CVC_C1e) at 50% coverage is the most cost-effective intervention in Southeast Asia, with an incremental cost-effectiveness of I$ 87 per HLY gained. At full coverage (95%), this combination intervention produces the highest effectiveness among all cervical cancer interventions. In eastern sub-Saharan Africa, vaccination against human papillomavirus (two doses) of 9–13-year-old girls (CVC_C1a) as an individual intervention, at 50% coverage is the most cost-effective intervention for cervical cancer, with an Incremental Cost-Effectiveness Ratio (ICER) of I$ 28 per HLY gained. For maximum health gain, this intervention then has to be progressively brought up to 95% coverage and combined with prevention of cervical cancer, by screening women aged 30–49 through visual inspection with acetic acid linked with timely treatment of pre-cancerous lesions (CVC_C1e).

For breast cancer, for both regions, treatment of breast cancer stages I and II with surgery ± systemic therapy (BRC_C2a) at 95% coverage is the most cost-effective intervention with an ICER of I$ 252 per HLY gained in Southeast Asia and I$ 113 per HLY gained in eastern sub-Saharan Africa. Screening with mammography (once every 2 years for women aged 50–69 years) linked with timely diagnosis and treatment (BRC_C2b) is less cost-effective, since mammography is a high-resource use technology. In addition, mammography requires a robust health infrastructure for a country to be able to sustain an organized population-based screening programme [36].

For colorectal cancer, for both regions, treatment of colorectal cancer, stages I and II, with surgery ± chemotherapy and radiotherapy (CRC_C3a) at 95% coverage is cost effective at I$ 238 per HLY gained in southeast Asia, and I$ 217 per HLY gained in eastern sub-Saharan Africa.

Overall, cervical cancer interventions are the most cost effective strategies among the studied interventions against cancer. Their favourable cost-effectiveness ratio arises from effective primary and/or secondary preventative strategies that effectively reduce the burden of disease at a low cost.

For all three cancers, basic palliative care is an essential element in cancer control that should be added at 95% coverage for optimal implementation.

**Fig. 1:**
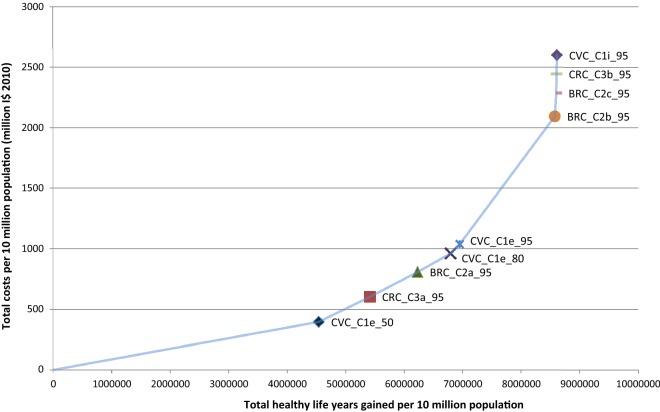
Cost effectiveness expansion path for Southeast Asia. Refer to Table 1 for interventions’ labels

**Fig. 2:**
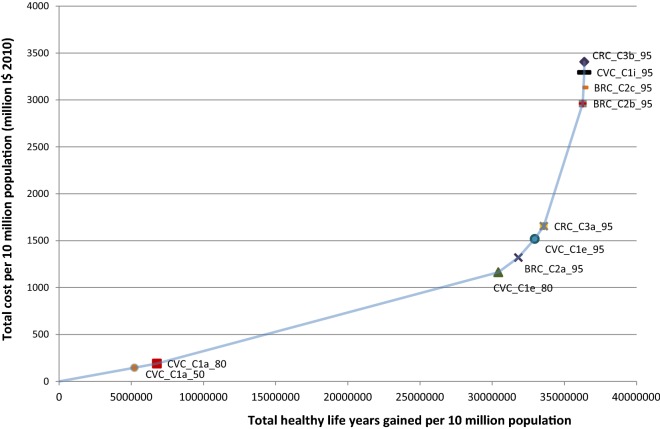
Cost effectiveness expansion path for Eastern sub-Saharan Africa. Refer to Table 1 for interventions’ labels

Figures 1 and 2 show the expansion path a decision maker would follow to achieve the maximum health gain in respectively, Southeast Asia and eastern sub-Saharan Africa.

If, with enough resources, all the interventions on the expansion path can be implemented, the budgetary allocation at full coverage across each of the three cancers would be as follows: in Southeast Asia: breast cancer, 56%; cervical cancer, 30%; colorectal cancer, 14%; in Eastern sub-Saharan Africa: Breast Cancer, 48%; Cervical Cancer, 45%; Colorectal Cancer, 7% (Table 4).

**Table 4:** Budgetary allocation among cancers for one country in Southeast Asia and Eastern sub-Saharan Africa for implementing the full expansion path at 95% coverage

Diseases	Total costs (per 10 million population)		Costs (%)	
	Southeast Asia	Eastern sub-Saharan Africa	Southeast Asia	Eastern sub-Saharan Africa
CVC	781,881,006	1,522,549,019	30	45
BRC	1,454,645,503	1,635,269,849	56	48
CRC	364,949,796	248,737,875	14	7
Total costs (per 10 million population)	2,601,476,305	3,406,556,743		
Total undiscounted HLY (per 10 million population)	8,611,060	36,378,783		
ACER	302.11	93.64		
Total discounted HLY (per 10 million population)	1,830,047	4,938,728		

## Discussion

### Principal findings

The burden of disease and economic impact of cancer are significant and increasing. Effective cancer control planning requires accurate data for planning, costing and implementation. This study assists policy makers in obtaining the best value for money for breast, cervical and colorectal cancer control by identifying the impact and costs of priority cancer control interventions as part of a comprehensive programme.

There are four principle findings in this study: (i) cancer prevention and control interventions are cost-effective and can significantly reduce the burden of disease globally; (ii) a step-wise approach to implementation that considers context-specific expansion paths can be utilized; (iii) interventions for early-stage cancers are generally more cost-effective than those for late-stage cancers; and (iv) palliative care programmes, which should be prioritized since it is considered as human right to health and recommended by the World Health Assembly [37–39], can be implemented at generally low cost.

Cancer and other noncommunicable diseases have received low priority, donor support and domestic resource allocation in low resource settings [40]. Contributing factors are the presumed high costs and low health impact of cancer interventions. This study highlights that cancer interventions are cost-effective and can be implemented in a comprehensive approach, in line with other NCD interventions as well as accepted communicable disease interventions [41]. Two interventions, in particular, were found to be highly cost-effective, exceeding an average cost-effectiveness ratio (ACER) threshold of less than I$ 100 per HLY. These interventions are the prevention of cervical cancer through HPV vaccination and the screening and treatment of pre-cancerous lesions. Critically, cost-effectiveness also depends on regional incidence—cervical cancer interventions are more cost effective in eastern sub-Saharan Africa than in Southeast Asia where incidence is lower.

Decision makers are faced with selecting priority cancer control interventions unique to their setting, recognizing the heterogeneity of cancer burden according to region and the differing capacity of health systems. Context-specific expansion paths can help inform decision makers by facilitating a step-wise approach to the implementation of cancer control interventions. For example, this study demonstrates the importance in cost-effectiveness terms of ramping up treatment for the early stages of disease before progressing to systematic cancer screening programmes, an approach which is moreover consistent with existing WHO guidance, based on programmatic considerations [20]. For example, in the expansion paths for both regions, treatment of breast cancer was found to be the most cost-effective breast cancer intervention, with compared to the null, an ICER of I$ 252 per HLY in southeast Asia (screening with mammography linked to timely diagnosis and treatment has an ICER of I$ 1048 per HLY). Thus, a step-wise approach provides additional evidence in support of the view that expanding treatment services should generally be considered before introducing population-level screening programmes.

This study also highlights the importance of diagnosing cancer early. Treatment for stage I colorectal cancer is approximately five times less expensive than treatment for stage II colorectal cancer. Furthermore, the impact of treatment is greater in stage I cancer as compared to stage II, III or IV [20, 42]. Accordingly, early diagnosis is particularly important to identify cancer at the stage when treatment is both more effective and less expensive. Cancer control strategies that facilitate early diagnosis can provide a significant return on investment [20]. In combination with the previous paragraph, this implies that treatment services need to be expanded then screening introduced, and only when early diagnosis is achieved will the best value for money in cancer control be obtained.

Finally, it is important to note that while palliative care is not as cost-effective as other cancer control intervention, it is an essential element of treatment, critical for human dignity, and it should be integrated into the continuum of care [38]. This study demonstrates that palliative care programmes can be introduced at a relatively low cost and with minimal health system requirements. This cancer control element should be prioritized, particularly given that more than 80% of the global population live in countries with low or non-existent access to adequate pain management [43].

### Strengths of the analysis

The methodology presented in this study uses a comprehensive, health systems approach to cost-effectiveness that considers diverse costs inputs including health workforce requirements, capital expenditures and consumables informed by existing WHO guidance in cancer control, programmatic monitoring and evaluation costs and service delivery costs such as false positive results associated with cancer screening. By identifying and costing all identifiable inputs, this analysis calculates total costs including the costs of health system factors required for effective implementation.

For example, breast cancer screening considers a mechanism for call and recall of the population, diagnostic tests, false positive findings including subsequent diagnosis and pathology, diagnostic tests including immunohistochemistry for hormone receptor testing, staging for select individuals found to have cancer, health workforce time for treatment, management of treatment related toxicities, inpatient and outpatient costs, surveillance after cancer treatment and monitoring and evaluation of screening. Inclusion of these elements results in a more robust and accurate model, as each of them can contribute significantly to the costs of cancer screening and treatment programmes [32, 44, 45].

Additionally, a review of effect sizes utilized in previous analysis based on the study performed by Disease Control Priority, Volume 3, Cancer was made to ensure selection of effect sizes and methodology are consistent with the best available evidence [46].

### Limitation of the analysis

There are six limitations to this analysis. First, while assumptions are based on best available evidence, there are gaps in high-quality evidence for cancer prevention and control interventions. For example, because of its relatively recent introduction to the market, there is limited longitudinal data on the durability of HPV vaccination and its effect in protection against cervical cancer. Another example is to quantify the impact of surgery for stage I breast cancer compared to the null state of no treatment available. As would be expected, there is no randomized controlled trial evaluating the impact of this intervention. To mitigate the impact of this limitation, assumptions were verified using available data such as historic publications and case series of patients who refuse treatment and/or aligned with previous assumptions in cancer cost-effectiveness studies; policy implications should be minor.

Second, there are insufficient studies for region- or country-specific variables. In this study, stage distribution, health workforce costs and programmatic costs were estimated based on available data. An assumption was made that the tumor biology/natural history of cancer was similar between settings. Additionally, the effect size of the intervention was used across all settings—that is, the impact of a particular intervention (e.g. vaccination, screening, treatment) was assumed to be equal in all setting. A literature review for region- or country-specific data was performed to address this limitation. However, there are limited data in low-resourced settings. Additional research is needed to develop regional specific inputs and variables; countries cannot generalize without regional or national epidemiologic data.

Third, the data used for the model were average regional estimates, as the scope of our work was generalized analyses of the cost-effectiveness of interventions. Application of the model to individual countries should consider more country-specific data inputs as available, and conduct sensitivity analyses around the input parameters for evaluating the impact of parameter variabilities on program decisions.

Fourth, the disability weights used were from the 2010 global burden diseases study. The development of the impact models began prior to the release of more recent disability weight data. As there has been minimal change in the disability weights for cancer stages in subsequent updates, and the costing baseline year is 2010, the authors were comfortable with continuing to use the 2010 estimates which fall well within the uncertainty bounds of latter estimates.

Fifth, various models have been used for costing cancer control programmes, such as the bottom-up or top-down method [47, 48]. Each strategy has advantages and disadvantages. In this study, the bottom-up approach was used, consistent with WHO-CHOICE methodology, allowing for comparison across diseases and settings. Furthermore, a thorough review of costing elements was considered to reduce any under-estimates. The GCEA is a standardized method for applying evidence to poor data settings where guidance is most needed. The tool has better use for priority setting than for budgeting. Results presented are intended to be indicative examples, rather than prescriptive packages or budgetary allocations for countries to implement. They must be evaluated prospectively to correlate with budgets or National Health Accounts.

Finally, regarding the health outcomes model used the transition parameters were grouped according to general cancer types. Different cancer subtypes, such as hormone receptor positive breast cancers, were not considered in this study. This model thus assumed that there is no significant heterogeneity in the cancer subtypes between different populations.

### Policy implications

The 2030 Agenda for Sustainable Development ushered in the era of universal health coverage (UHC) as a global priority. In order to achieve targets related to UHC, including financial protection, and reduce premature mortality from NCDs, a basic package of cancer services must be identified. Domestic, bilateral and multilateral funding should be channeled towards evidence-based, cost-effective interventions for cancer prevention and control, thereby avoiding unnecessary expenditure on high-cost interventions, medicines and technologies that yield less health benefit for populations [49]. This study provides the foundation for region-specific data to identify the most cost-effective cancer interventions that can be considered for inclusion in a basic package of cancer services.

## Conclusion

This study presents the new WHO-CHOICE cost-effectiveness results for three priority cancers, utilizing region-specific data to support decision-making based on epidemiologic profile, regional costs, and health system capacity. The results demonstrate that cancer prevention and control interventions are cost-effective and can be implemented through a step-wise approach to achieve maximum health benefits. As the global community moves toward universal health coverage, this analysis can support decision makers in identifying a core package of cancer services, ensuring treatment and palliative care for all. Results are provided at regional level, an obvious contextualization is necessary for an individual country level implementation [50].

## Additional files

**Additional file 1.** State-transition (Markov model) cohort simulation model for estimation of health outcomes presented in the main manuscript.

**Additional file 2.** Effect sizes, costing assumptions and detailed results per region.

## Abbreviations

ACER: average cost-effectiveness ratio
AFR: Africa region
CHOICE: choosing interventions that are cost-effective
CIS: carcinoma in situ
DWs: disability weights
GBD: Global Burden of Disease
GCEA: Generalized Cost Effectiveness Analysis
HER2: human epidermal growth factor receptor 2
HLYs: healthy life years
HPV: human papillomavirus
ICER: incremental cost-effectiveness ratio
IHME: Institute for Health Metrics and Evaluation
LMIC: low and middle income countries
MDGs: millennium development goals
MSH: management sciences for health
NCDs: noncommunicable diseases
SEAR: South-East Asia region
SDGs: sustainable development goals
UHC: universal health coverage
WHO: World Health Organization
