# Supplementary material for: Cost-effective interventions for breast cancer, cervical cancer, and colorectal cancer: new results from WHO-CHOICE
Source: Cost Eff Resour Alloc. 2018 Oct 29;16:38. doi: 10.1186/s12962-018-0157-0 (PMC6206923; doi:10.1186/s12962-018-0157-0)
Supplement: Supplementary file 1 — Additional file 1. State-transition (Markov model) cohort simulation model for estimation of health outcomes presented in the main manuscript. [file 12962_2018_157_MOESM1_ESM.docx]

# **Cost-effective interventions for breast cancer, cervical cancer, and colorectal cancer: new results from WHO-CHOICE**

# Additional file 1: State-transition (Markov model) cohort simulation model for estimation of health outcomes presented in the main manuscript

## Overview

Health outcomes of the disease and the impact of alternative interventions for breast cancer, cervical cancer, and colorectal cancer were evaluated using a deterministic state-transition (Markov model) cohort simulation. The general structure of the model is described in the main manuscript. Here we present the mathematical structure of the simulation using breast cancer as an example. The states and transitions of the Markov model are depicted as flow diagram in Figures S1 (breast cancer), S2-S4 (cervical cancer), and S5 (colorectal cancer).

## Mathematical structure of the simulation using breast cancer as an example

Let, Ζ$=\left\{ H,UC1,UC2,UC3,UC4, C1,C2,C3,C4 \right\}$ be the state space, a set of mutually exclusive collectively exhaustive states, of the Markov model containing healthy (H), preclinical (UC1,UC2,UC3,U4), and clinical stages (C1,C2,C3,C4) of breast cancer (see Figure S1 for reference), and

$\mathbb{Q}$ be a matrix of transition rates (per person year) between states.

|  |  | $H$ | $UC1$ | $UC2$ | $UC3$ | $UC4$ | $C1$ | $C2$ | $C3$ | $C4$ |
| --- | --- | --- | --- | --- | --- | --- | --- | --- | --- | --- |
|  | $H$ | $-y-m$ | $y$ |  |  |  |  |  |  |  |
|  | $UC1$ |  | $-p1-d1-m$ | $p1$ |  |  | $d1$ |  |  |  |
|  | $UC2$ |  |  | $-p2-d2-m$ | $p2$ |  |  | $d2$ |  |  |
|  | $UC3$ |  |  |  | $-p3-d3-m$ | $p3$ |  |  | $d3$ |  |
| $\mathbb{Q}=$ | $UC4$ |  |  |  |  | $-d4-m5$ |  |  |  | $d4$ |
|  | $C1$ |  |  |  |  |  | $-m1$ |  |  |  |
|  | $C2$ |  |  |  |  |  |  | $-m2$ |  |  |
|  | $C3$ |  |  |  |  |  |  |  | $-m3$ |  |
|  | $C4$ |  |  |  |  |  |  |  |  | $-m4$ |

*Note: empty cells =0*

where, (see Figure S1 for reference of notations)

$y$ are the disease onset rates

$p1, p2, p3$are the progression rates

$d1, d2, d3, d4$are the diagnostic rates

$m$are the disease-free mortalities, and

$m1, m2, m3, m4$are the disease mortalities with treatment,

$m5$ are the disease mortality rates without treatment

To simulate the population we use a set of first-order differential equations given by

${\rho'}_{t+1}= {\rho'}_{t}+ \rho_{t}\mathbb{Q}\Delta t$

where,

$\rho_{t}=[H,UC1,UC2,UC3,UC4, C1,C2,C3,C4]$, is a vector with each element equal to the number of people in that state (denoted in the vector) at time $t$

$\rho_{t}'$ is the transpose of the vector $\rho_{t}$

$\Delta t$ is a small time-step

## Simulation steps

Initialization:

- Set $t=$ base year of simulation.
- $\Delta t=$ suitably small time-step
- For each age-group in the simulation, set $\rho_{t}$ as population in base year of simulation

Repeat below steps until $t=$ final year of simulation

1. For each age-group in the simulation, apply ${\rho'}_{t+1}= {\rho'}_{t}+ \rho_{t}\mathbb{Q}\Delta t$, taking age-specific rates for elements of $\mathbb{Q}$ where applicable
2. For the first age-group in the simulation, increment $\rho_{t}\left( 1 \right)=\rho_{t}\left( 1 \right)+births$
3. Increment $t$=$t+\Delta t$

Similar structures were developed for cervical cancer and colorectal cancer. All transition rates of the Markov models were assumed static (except for HPV transmission rates), i.e., we do not model changes in cancer risk in the population due to changes in factors such as lifestyle or environment. In the case of cervical cancer simulation, we dynamically estimate HPV transmission rates over time to capture the changes in risk from interventions such as vaccination.

## Dynamic estimation of HPV transmission rates in the cervical cancer simulation

The cervical cancer simulation dynamically estimates HPV transmission rates in men and women over time using

$\bar{r}_{i}=\alpha\bar{\partial}.\left( \bar{\mathbb{M}}\beta_{i} \right)\left( 1-\bar{c}_{i} \right); r_{i}=t\partial.(\mathbb{M}\bar{\beta}_{i})(1-c_{i})$ where,

$\bar{r}_{i} , r_{i}$ are the age-based column-vectors of HPV infection rates for HPV type $i$ in men and women, respectively,

$\bar{r}_{i}$ =$\left\{ \bar{r}_{16-18},\bar{r}_{high-risk},\bar{r}_{low-risk} \right\}\equiv${r32, r33, or r34} in Figure S4,

$r_{i}= \left\{ r_{16-18},r_{high-risk},r_{low-risk} \right\}\equiv$ {r1, r23, or r8} in Figure S2,

$\bar{\partial}$ and $\partial$ are the age-based column vectors for partner exposure-rates, which we assume are inclusive of multiple sexual parameters such as partner turn-over rate, and number of sexual exposures not 100% protected by condoms, for men and women, respectively,

$\bar{\mathbb{M}}$and $\mathbb{M}$ are matrices representing age-mixing of sexual partnerships for men and women, respectively; each element $m_{jk}\in\bar{\mathbb{M}}$ representing the probability that a man in age $j$ has a partnership with a woman of age $k$, and $m_{jk}\mathbb{\in M}$ representing the probability that a woman in age $j$ has a partnership with a man of age $k$, each row adding to 1,

$\bar{\beta}_{i}$and$\beta_{i}$ are the age-based column vectors of prevalence of HPV-type $i$ in men and women, respectively,

$\alpha$ is the probability of transmission per infected-susceptible contact (t $\approx1$ for HPV), and

$\bar{c}_{i},c_{i}$ are the coverage of vaccination for HPV type $i$ in men and women, respectively, which is 0 in the base case.

We assume that $\bar{\partial}, \partial, \bar{\mathbb{M}}\mathrm{and}\mathbb{M}$are available or can be estimated through other sexual behavior data available from national surveys, here we estimated them using partnership age differences from the Demographics and Health Surveys (DHS). $\bar{\beta}_{i}$and$\beta_{i}$ are estimated dynamically in the simulation using $\rho_{t}$.

## Markov model transition rate estimates and data sources

The transition rates for the natural progression of cancer are presented in Tables S1-S2 (breast cancer), S3-S4 (cervical cancer), and S5-S6 (colorectal cancer). We assumed that disease onset rates, i.e., transitions from healthy to first stage of disease, and diagnostic rates, i.e., transitions from preclinical to clinical stages of cancer, vary by population, these rates are presented in Table S2, S4, S6 for 2 world regions. These rates were estimated using a newly developed methodology that is presented elsewhere [1]. We assume that progression and regression rates between cancer stages do not vary by population, these rates and data sources are presented in Tables S1, S3, S5.

## Figure S1: State-transition model for breast cancer in women


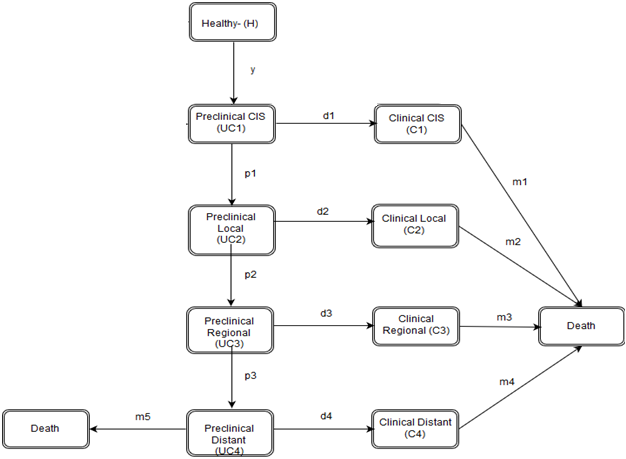


## Table S1: Breast cancer- Transition rates for natural disease progression for breast cancer state-transition model in Figure S1

| Parameters | Value |  | Source |
| --- | --- | --- | --- |
|  |  |  | [2], [3], [4] |
| Progression rates |  |  |  |
| In-situ to Local ($p_{1}$) | $0.19$ |  |  |
| Local to Regional ($p_{2}$) | 0.33 |  |  |
| Regional to Distant ($p_{3}$) | $0.43$ |  |  |
| Annual mortality rate (per person year) with treatment by stage at diagnosis | | |  |
| In-situ ($m_{1}$) | 0.01 |  |  |
| Local ($m_{2}$) | 0.02 |  |  |
| Regional ($m_{3}$) | 0.08 |  |  |
| Distant ($m_{4}$) | 0.27 |  |  |
|  |  |  |  |

## Table S2: Breast cancer- Population-specific natural disease onset rates and diagnostic rates for breast cancer state-transition model in Figure S1

| Age group | | | | | Eastern  Sub-Saharan Africa | | | | |  | | | Southeast Asia | | |  |
| --- | --- | --- | --- | --- | --- | --- | --- | --- | --- | --- | --- | --- | --- | --- | --- | --- |
|  | | | | | | | | | | | | | | |  | |
| Onset rates of in-situ (y)(per 1000 persons per year) | | | | | | | | | | | | | | |  | |
| Age Groups |  | Eastern Sub-Saharan Africa | | | | | |  | | | Southeast Asia | | | | | |
|  | | |  |  | |  |  | |  | | |  | |  |  | |
| 15_19 | | |  |  | | 0.07 |  | |  | | |  | | 0.07 |  | |
| 20_24 | | |  |  | | 0.18 |  | |  | | |  | | 0.17 |  | |
| 25_29 | | |  |  | | 0.35 |  | |  | | |  | | 0.34 |  | |
| 30_39 | | |  |  | | 0.57 |  | |  | | |  | | 0.58 |  | |
| 40_49 | | |  |  | | 1.45 |  | |  | | |  | | 1.67 |  | |
| 50_59 | | |  |  | | 2.44 |  | |  | | |  | | 2.96 |  | |
| 60_69 | | |  |  | | 3.57 |  | |  | | |  | | 3.82 |  | |
|  | | |  |  | | | | |  | | |  | |  |  | |
| Diagnosis rates (per year) | | | | | | | | | | | | | |  |  | |
| Age Groups |  | Eastern Sub-Saharan Africa | | | | | |  | | | Southeast Asia | | | | | |
|  | | |  | Local | | Regional | Distant | |  | | | Local | | Regional | Distant | |
| 15_19 | | |  | 0.38 | | 0.83 | 1.00 | |  | | | 0.49 | | 0.95 | 1.00 | |
| 20_24 | | |  | 0.33 | | 0.73 | 0.87 | |  | | | 0.39 | | 0.76 | 0.80 | |
| 25_29 | | |  | 0.20 | | 0.45 | 0.53 | |  | | | 0.24 | | 0.46 | 0.49 | |
| 30_39 | | |  | 0.24 | | 0.53 | 0.63 | |  | | | 0.29 | | 0.56 | 0.59 | |
| 40_49 | | |  | 0.20 | | 0.43 | 0.52 | |  | | | 0.25 | | 0.49 | 0.51 | |
| 50_59 | | |  | 0.09 | | 0.21 | 0.25 | |  | | | 0.12 | | 0.23 | 0.25 | |
| 60_69 | | |  | 0.05 | | 0.11 | 0.13 | |  | | | 0.06 | | 0.11 | 0.12 | |
|  | | |  |  | |  |  | |  | | |  | |  |  | |

## Figure S2: Overview of HPV and cervical cancer state-transition model

Disease Free Women

Disease Free Men

Module for HPV infection and cervical cancer in women (Figure S3)

Module for HPV infection in men (Figure S4)

Transmission model

## Figure S3: State-transitions model for HPV infection and cervical cancer in women


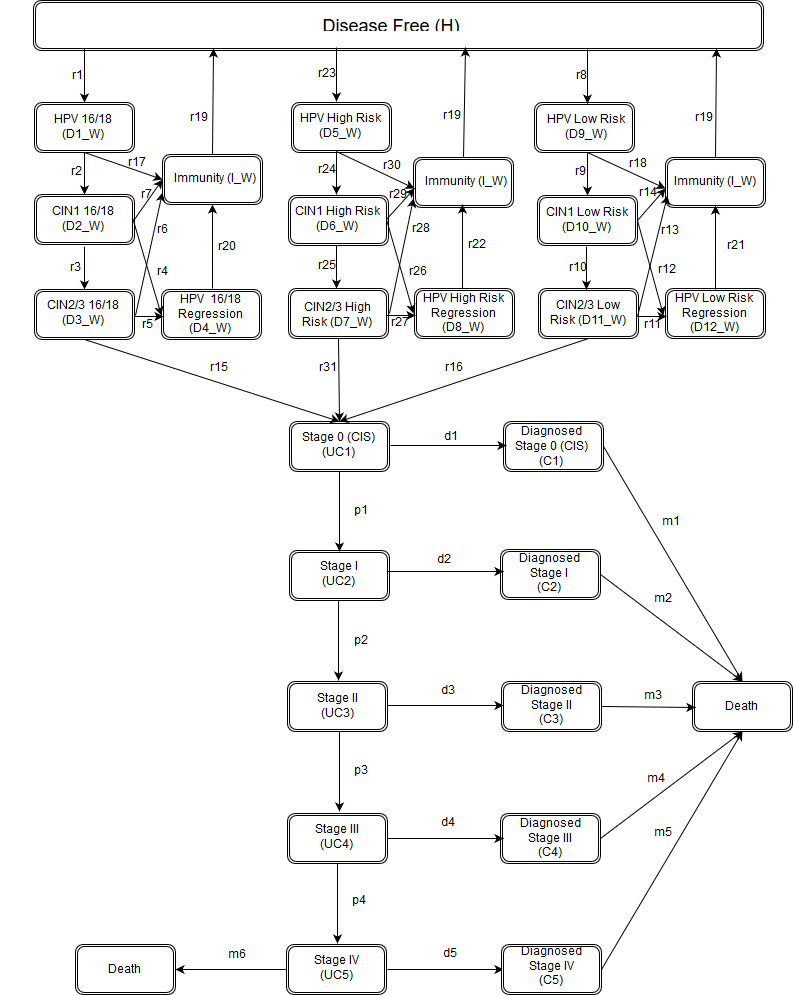


## Figure S4: State-transition model for HPV infection in men


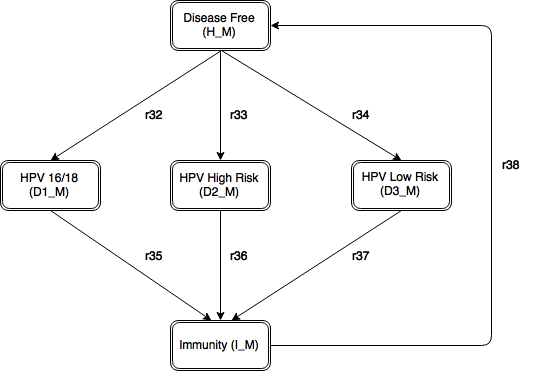


## Table S3: HPV and Cervical cancer- Transition rates for natural disease progression for cervical cancer state-transition model in Figures S2- S4

| Parameters | Values | | | | |  | | | Source | | | |  |  |
| --- | --- | --- | --- | --- | --- | --- | --- | --- | --- | --- | --- | --- | --- | --- |
|  | | |  | | | | | | |  | |  | |  |
| PARAMETERS FOR WOMEN | | | HPV types | | | | | | | [5], [6] | |  | |  |
|  |  |  | 16/18 | High risk^[[1]](#footnote-1)^ | | | Low risk | | |  | |  | |  |
| Transition rates in pre-cancer stages 2  (per person year) 2 | | | |  | | |  | | |  | |  | |  |
|  | | |  |  | | |  | | |  | |  | |  |
| HPV to CIN 1 ($r_{2}, r_{9} ,r_{24}$) | | | 0.0931 | 0.0931 | | | 0.0568 | | |  | |  | |  |
| CIN 1 to CIN 2/3 ($r_{3}, r_{10} ,r_{25}$) | | | 0.2107 | 0.2107 | | | 0.0921 | | |  | |  | |  |
| CIN 2/3 to CIS ($r_{15}, r_{16} ,r_{31}$) | | |  |  | | |  | | |  | |  | |  |
| 1-30 years | | | 0.0292 | 0.0292 | | | 0.007 | | |  | |  | |  |
| 30–39 years | | | 0.0506 | 0.0506 | | | 0.014 | | |  | |  | |  |
| 40–49 years | | | 0.1344 | 0.1344 | | | 0.0221 | | |  | |  | |  |
| 50-100 years | | | 0.1952 | 0.1952 | | | 0.0445 | | |  | |  | |  |
| HPV to Immunity ($r_{17}, r_{18}, r_{30}$) | | | 0.0363 | 0.0363 | | | 0.0363 | | |  | |  | |  |
| CIN 1 to Immunity ($r_{7}, r_{14}, r_{29}$) | | | 0.1188 | 0.1188 | | | 0.1059 | | |  | |  | |  |
| CIN 1 to Regression ($r_{4}, r_{12} ,r_{26}$) | | | 0.1188 | 0.1188 | | | 0.1059 | | |  | |  | |  |
| CIN 2 to Immunity ($r_{6}, r_{13} ,r_{28}$) | | | 0.0171 | 0.0171 | | | 0.0704 | | |  | |  | |  |
| CIN 2 to Regression ($r_{5}, r_{11} ,r_{27}$) | | | 0.0171 | 0.0171 | | | 0.0704 | | |  | |  | |  |
| Regression to Immunity ($r_{20},r_{22},r_{21}$) | | | 0.0363 | 0.0363 | | | 0.0363 | | |  | |  | |  |
| Immunity to Disease Free ($r_{19}$) | | | 0.1000 | 0.1000 | | | 0.1000 | | |  | |  | |  |
|  | | | | | | | | | |  | |  | |  |
| Transition rates in preclinical stages (per person year) | | | | | | | | | | [6] | |  | |  |
|  | | | | | | | | | |  | |  | |  |
| Stage 0 (CIS) to Stage I ($p_{1}$) | | |  | All Types | | | | | |  | |  | |  |
| 1-34 years | | |  | 0.03 | | |  | | |  | |  | |  |
| 35–54 years | | |  | 0.273 | | |  | | |  | |  | |  |
| 55–61 years | | |  | 1.185 | | |  | | |  | |  | |  |
| 62–100 years | | |  | 5.290 | | |  | | |  | |  | |  |
| Stage I to Stage II ($p_{2}$) | | |  | 0.310 | | |  | | |  | |  | |  |
| Stage II to Stage III ($p_{3}$) | | |  | 0.332 | | |  | | |  | |  | |  |
| Stage III to Stage IV ($p_{4}$) | | |  | 0.485 | | |  | | |  | |  | |  |
|  | | |  |  | | |  | | |  | |  | |  |
| Annual mortality rate with treatment (per person year) | | | | | | | | | | | | [6] | |  |
|  | | | | | | | | | | | |  | |  |
| Stage I ($m_{2}$) | | |  | 0.027 | | |  | | |  | |  | |  |
| Stage II ($m_{3}$) | | |  | 0.062 | | |  | | |  | |  | |  |
| Stage III ($m_{4})$ | | |  | 0.167 | | |  | | |  | |  | |  |
| Stage IV ($m_{5}$) | | |  | 0.316 | | |  | | |  | |  | |  |
|  | | |  |  | | |  | | |  | |  | |  |
| PARAMETERS FOR MEN | |  | | |  | | |  | | |  | | | |
|  | |  | | |  | | |  | | |  | | | |
|  | | 16/18 | | | High risk | | | Low risk | | |  | | | |
| Transition rates in men (per person-year) | | | | |  | | |  | | | [6] | | | |
|  | |  | | |  | | |  | | |  | | | |
| HPV to Immunity ($r_{35},r_{36},r_{37}$) | | 0.0363 | | | 0.0363 | | | 0.0363 | | |  | | | |
| Immunity to Disease Free ($r_{38}$) | | 0.1 | | | 0.1 | | | 0.1 | | |  | | | |
|  | |  | | |  | | |  | | |  | | | |

^1^ All high-risk types of HPV other than type 16/18.

^2^ 6-months probabilities from [4] have been converted to annual rates using $-[ln(1-p)]/t$ where $p$ is the probability and $t$ is the time in years.

## Table S4: Cervical cancer- Population-specific natural disease onset rates and diagnostic rates for HPV and cervical cancer state-transition model in Figures S2-S4)

| Age group | | | Eastern  Sub-Saharan Africa | | | | | | | | | | |  | | | | | Southeast Asia | | | | | | |  |  |
| --- | --- | --- | --- | --- | --- | --- | --- | --- | --- | --- | --- | --- | --- | --- | --- | --- | --- | --- | --- | --- | --- | --- | --- | --- | --- | --- | --- |
|  | | | | | | | | | | | | | | | | | | | | | |  | | | | |  |
| PARAMETERS FOR WOMEN | | | | | | | | | | | | | | | | | | | | | |  | | | | |  |
|  | | | | | | | | | | | | | | | | | | | | | |  | | | | |  |
| Onset rates of HPV (per 1000 women per year) | | | | | | | | | | | | | | | | | | | | | |  | | | | |  |
| Age Groups | Eastern Sub-Saharan Africa | | | | | | | | | | | | Southeast Asia | | | | | | | | | | | | | |  |
|  | 16/18 | | | | | High risk | | | Low risk | | | | 16/18 | | | | | | | High risk | | | | Low risk | | |  |
|  |  | | | | |  | | |  | | | |  | | | | | | |  | | | |  | | |  |
| 15-19 | 8.20E-03 | | | | | 1.80E-03 | | | 6.82E-03 | | | | 7.20E-03 | | | | | | | 2.80E-03 | | | | 4.63E-03 | | |  |
| 20-24 | 130.14 | | | | | 28.57 | | | 11.66 | | | | 47.11 | | | | | | | 18.32 | | | | 4.69 | | |  |
| 25-29 | 46.57 | | | | | 10.22 | | | 7.01 | | | | 16.02 | | | | | | | 6.23 | | | | 2.1 | | |  |
| 30-39 | 49.27 | | | | | 10.81 | | | 6.54 | | | | 14.11 | | | | | | | 5.49 | | | | 1.68 | | |  |
| 40-49 | 39.38 | | | | | 8.64 | | | 3.13 | | | | 8.01 | | | | | | | 3.12 | | | | 0.58 | | |  |
| 50-59 | 38.5 | | | | | 8.45 | | | 2.71 | | | | 7.95 | | | | | | | 3.09 | | | | 0.49 | | |  |
| 60-69 | 47.68 | | | | | 10.47 | | | 3.15 | | | | 9.04 | | | | | | | 3.51 | | | | 0.54 | | |  |
|  |  | | | | |  | | |  | | | |  | | | | | | |  | | | |  | | |  |
| Diagnosis rates of cervical cancer (per 1000 person years among women in pre-clinical stages) | | | | | | | | | | | | | | | | | | | | | |  | | | | |  |
| Age Groups | Eastern Sub-Saharan Africa | | | | | | | | | | | | Southeast Asia | | | | | | | | | | | | | |  |
|  | Stage I | | | Stage II | | | Stage III | | | Stage IV | | | Stage I | | | Stage II | | | | | Stage III | | | | Stage IV | |  |
| 15-19 | 171.15 | | | 375.31 | | | 838.63 | | | 1000 | | | 193.28 | | | 554.62 | | | | | 949.58 | | | | 1000 | |  |
| 20-24 | 171.15 | | | 375.31 | | | 838.63 | | | 1000 | | | 193.28 | | | 554.62 | | | | | 949.58 | | | | 1000 | |  |
| 25-29 | 161.68 | | | 354.54 | | | 792.23 | | | 944.67 | | | 188.91 | | | 542.09 | | | | | 928.12 | | | | 977.4 | |  |
| 30-39 | 44.5 | | | 97.58 | | | 218.05 | | | 260 | | | 42.29 | | | 121.35 | | | | | 207.76 | | | | 218.8 | |  |
| 40-49 | 30.06 | | | 65.91 | | | 147.29 | | | 175.63 | | | 26.69 | | | 76.6 | | | | | 131.15 | | | | 138.11 | |  |
| 50-59 | 11.94 | | | 26.19 | | | 58.52 | | | 69.78 | | | 8.97 | | | 25.74 | | | | | 44.06 | | | | 46.4 | |  |
| 60-69 | 9.06 | | | 19.87 | | | 44.41 | | | 52.95 | | | 6.89 | | | 19.76 | | | | | 33.84 | | | | 35.63 | |  |
|  |  | | |  | | |  | | |  | | |  | | |  | | | | |  | | | |  | |  |
| PARAMETERS FOR MEN | | | | | | | |  | | | |  | | | | |  | | | | | | | | | | |
|  | | | | | | | | | | | | | | |  | | | | | | | | | | | | |
| Onset rates of HPV (per 1000 person years) | | | | | | | | | | | | | | |  | | | | | | | | | | | | |
|  | | Eastern Sub-Saharan Africa | | | | | | | | | Southeast Asia | | | | | | | | | | | | | | | | |
|  | |  | | | | | | | | |  | | | | | | | | | | | | | | | | |
| Age group | | 16/18 | | | High risk | | | Low risk | | | 16/18 | | | | | | | High risk | | | | | Low risk | | | | |
|  | |  | | |  | | |  | | |  | | | | | | |  | | | | |  | | | | |
| 15-19 | | 0 | | | 0 | | | 0 | | | 0 | | | | | | | 0 | | | | | 0 | | | | |
| 20-24 | | 22.53 | | | 4.95 | | | 2.26 | | | 22.34 | | | | | | | 8.69 | | | | | 2.32 | | | | |
| 25-29 | | 30.37 | | | 6.67 | | | 3.14 | | | 9.63 | | | | | | | 3.75 | | | | | 1.01 | | | | |
| 30-39 | | 49.85 | | | 10.94 | | | 5.48 | | | 3.57 | | | | | | | 1.39 | | | | | 0.38 | | | | |
| 40-49 | | 39.73 | | | 8.72 | | | 4.27 | | | 0.27 | | | | | | | 0.1 | | | | | 0.03 | | | | |
| 50-59 | | 30.21 | | | 6.63 | | | 2.8 | | | 1.83E-02 | | | | | | | 7.12E-03 | | | | | 1.62E-03 | | | | |
| 60-69 | | 24.5 | | | 5.38 | | | 1.99 | | | 1.39E-03 | | | | | | | 5.40E-04 | | | | | 1.10E-04 | | | | |
|  | |  | | |  | | |  | | |  | | | | | | |  | | | | |  | | | | |

## Figure S5: State-transition model for colorectal pre-cancerous polyps and cancer


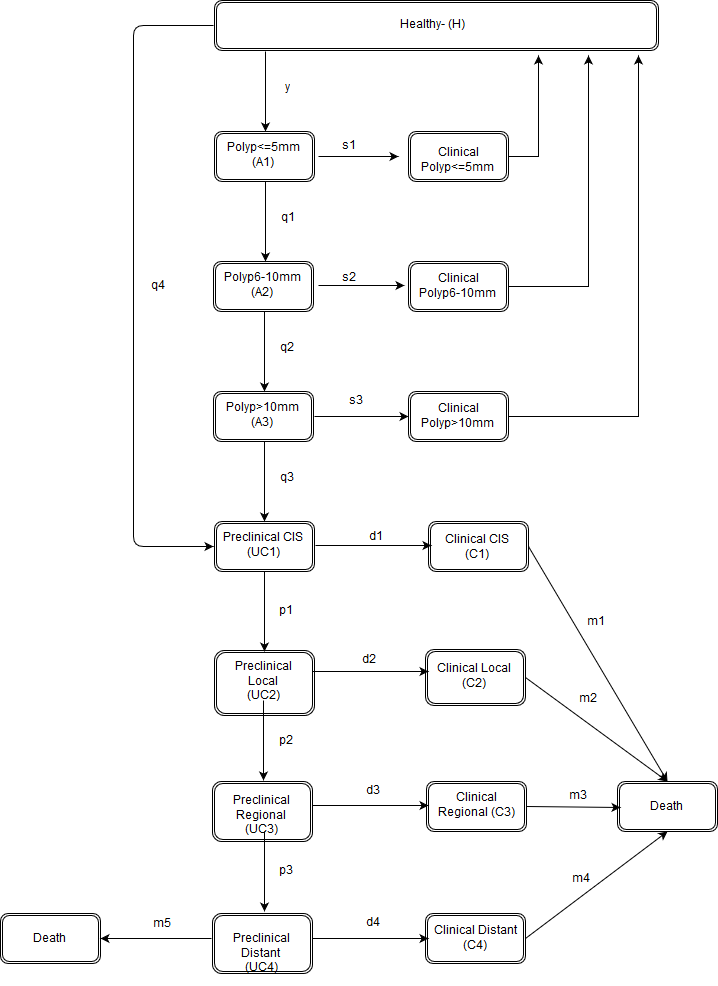


## Table S5: Colorectal cancer- Transition rates for natural disease progression for colorectal cancer state-transition model in Figure S5

| Parameters | Value |  | Source |
| --- | --- | --- | --- |
| Progression rates (per person year) |  |  |  |
| PolypLT5mm to Polyp6to10mm (q­­_1_) | 0.021 |  | [7], [8], [9] |
| PolypLT10mm to PolypGT10mm (q­­_2_) | 0.057 |  |  |
| PolypLT10mm to Preclinical 0 (q­­_3_) | 0.063 |  |  |
|  |  |  |  |
| In-situ to Local (p_1_) | 0.29 |  | [9], [10], [11], [12], [13], [14] |
| Local to Regional (p_2_) | 0.34 |  |  |
| Regional to Distant (p_3_) | 0.64 |  |  |
|  |  |  |  |
| Proportion of cancers from de novo carcinoma (q4) | 23% |  | [7], [15] |
|  |  |  |  |
| Annual mortality rate (per person year) with treatment |  |  | [9], [10], [11], [12], [13], [14] |
| In-situ ($m_{1}$) | 0.01 |  |  |
| Local ($m_{2}$) | 0.01 |  |  |
| Regional ($m_{3}$) | 0.05 |  |  |
| Distant ($m_{4}$) | 0.57 |  |  |
|  |  |  |  |

## Table S6: Colorectal cancer- Population-specific natural disease onset rates and diagnostic rates for colorectal cancer state-transition model in Figure S5

| Age groups | | | | Eastern  Sub-Saharan Africa | | |  | | Southeast Asia | | |  |
| --- | --- | --- | --- | --- | --- | --- | --- | --- | --- | --- | --- | --- |
|  | | | | | | | | | | |  | |
| Rate of adenoma polyp onset (y)(per 1000 person-years) | | | | | | | | | | |  | |
|  |  |  | | | |  | |  | | | | |
| Age Groups |  | Eastern Sub-Saharan Africa | | | |  | | Southeast Asia | | | | |
| 20_24 |  |  | 1.6 | |  |  | |  | | 3.4 |  | |
| 25_29 |  |  | 2.1 | |  |  | |  | | 4.4 |  | |
| 30_39 |  |  | 2.1 | |  |  | |  | | 4.5 |  | |
| 40_49 |  |  | 2.1 | |  |  | |  | | 4.7 |  | |
| 50_59 |  |  | 2.2 | |  |  | |  | | 4.9 |  | |
| 60_69 |  |  | 2.2 | |  |  | |  | | 5.2 |  | |
| 70_79 |  |  | 2.2 | |  |  | |  | | 5.4 |  | |
|  |  |  | | | |  | |  | |  |  | |
| Diagnostic rates (per person year) (rates for in-situ (d1) are zero) | | | | | | | | | |  |  | |
| Age Groups |  | Eastern Sub-Saharan Africa | | | |  | | Southeast Asia | | | | |
|  |  | Local(d2) | Regional(d3) | | Distant(d4) |  | | Local(d2) | | Regional(d3) | Distant(d4) | |
| 15_19 |  | 0.01 | 0.06 | | 0.10 |  | | 0.01 | | 0.06 | 0.10 | |
| 20_24 |  | 0.01 | 0.12 | | 0.19 |  | | 0.01 | | 0.07 | 0.11 | |
| 25_29 |  | 0.03 | 0.39 | | 0.60 |  | | 0.01 | | 0.14 | 0.22 | |
| 30_39 |  | 0.04 | 0.47 | | 0.72 |  | | 0.03 | | 0.31 | 0.48 | |
| 40_49 |  | 0.05 | 0.62 | | 0.96 |  | | 0.05 | | 0.56 | 0.86 | |
| 50_59 |  | 0.06 | 0.64 | | 1.00 |  | | 0.06 | | 0.64 | 1.00 | |
| 60_69 |  | 0.05 | 0.63 | | 0.98 |  | | 0.06 | | 0.64 | 1.00 | |
| 70_79 |  | 0.04 | 0.43 | | 0.67 |  | | 0.05 | | 0.58 | 0.90 | |
|  |  |  |  | |  |  | |  | |  |  | |
|  |  |  |  | |  |  | |  | |  |  | |
|  |  |  |  | |  |  | |  | |  |  | |

## References

[1] Gopalappa C, Guo J, Meckoni P, Munkhbat B, Pretorius C, Lauer J, Ilbawi A, Bertram M. A two-step Markov processes approach for parameterization of cancer state-transition models for low- and middle- income countries. Medical Decision Making. In press.

[2] Okonkwo Q, Draisma G, der Kinderen A, Brown M, de Koning H. Breast cancer screening policies in developing countries: a cost-effectiveness analysis for India. Journal of the National Cancer Institute. 2008; 100(18): 1290-1300.

[3] Zelle S, Baltussen R, Otten J, Heijnsdijk E, van Schoor G, Broeders M. Predicting the stage shift as a result of breast cancer screening in low- and middle-income countries: a proof of concept. Journal of Medicine Screening. 2014; 22(1): 8-19.

[4] Groot M, Baltussen R, Uyl-de Groot C, Anderson B, Hortobágyi G. Costs and health effects of breast cancer interventions in epidemiologically different regions of Africa, North America, and Asia. The Breast Journal. 2006; 12(s1): S81-S90.

[5] Insinga R, Dasbach E, Elbasha E. Epidemiologic natural history and clinical management of Human Papillomavirus (HPV) Disease: a critical and systematic review of the literature in the development of an HPV dynamic transmission model. BMC Infectious diseases. 2009; 9:119.

[6] Goldie S, Grima D, Kohli M, Wright T, Weinstein M, Franco E. A comprehensive natural history model of HPV infection and cervical cancer to estimate the clinical impact of a prophylactic HPV‐16/18 vaccine. International Journal of Cancer. 2003; 106(6): 896-904.

[7] Chen C, Yen M, Wang W, Wong J, Chen T. A case-cohort study for the disease natural history of adenoma-carcinoma and de novo carcinoma and surveillance of colon and rectum after polypectomy: implication for efficacy of colonoscopy. British Journal of Cancer. 2003; 88: 1866-1873.

[8] Leshno M, Halpern Z, Arber N. Cost-effectiveness of colorectal cancer screening in the average risk population. Health Care Management Science. 2003; 6(3):165-74.

[9] Frazier A, Colditz G, Fuchs C, Kuntz K. Cost-effectiveness of screening for colorectal cancer in the general population. JAMA. 2000; 284(15): 1954-61.

[10] Liu C, Chen W, Kung P, Chiu C, Wang Y, Shieh S, Tsai W. Characteristics, survival, and related factors of newly diagnosed colorectal cancer patients refusing cancer treatments under a universal health insurance program. BMC Cancer. 2014;14:446.

[11] Kuntz K, Lansdorp-Vogelaar I, Rutter C, Knudsen A, van Ballegooijen M, Savarino J, Feuer E, Zauber A. A systematic comparison of microsimulation models of colorectal cancer: the role of assumptions about adenoma progression. Medical Decision Making. 2011; 31(4): 530-9.

[12] Wu G, Wang Y, Yen A, Wong J, Lai H, Warwick J, Chen T. Cost-effectiveness analysis of colorectal cancer screening with stool DNA testing in intermediate-incidence countries. BMC Cancer. 2006; 6:136.

[13] Chadder J, Dewar R, Schack L, Nishri D, Niu J, Lockwood G. A first look at relative survival by stage for colorectal and lung cancers in Canada. Current Oncology. 2016; 23(2):119-124.

[14] National Cancer Intelligence Network (NCIN). Colorectal Cancer Survival by Stage - NCIN Data Briefing. 2009. <http://www.ncin.org.uk/publications/data_briefings/colorectal_cancer_survival_by_stage> Accessed 13 May 2017.

[15] Goto H, Oda Y., Murakami Y, Tanaka T, Hasuda K, Goto S, Sasaki Y, Sakisaka S, Hattori M. Proportion of de novo cancers among colorectal cancers in Japan. Gastroenterology. 2006; 131(1):40-6.

1. [↑](#footnote-ref-1)
