# Supplementary material for: Cost-effective interventions for breast cancer, cervical cancer, and colorectal cancer: new results from WHO-CHOICE
Source: Cost Eff Resour Alloc. 2018 Oct 29;16:38. doi: 10.1186/s12962-018-0157-0 (PMC6206923; doi:10.1186/s12962-018-0157-0)
Supplement: Supplementary file 2 — Additional file 2. Effect sizes, costing assumptions and detailed results per region. [file 12962_2018_157_MOESM2_ESM.docx]

# Cost-effective interventions for breast cancer, cervical cancer, and colorectal cancer: new results from WHO-CHOICE

# Additional file 2: Effect sizes, costing assumptions and detailed results per region

### Table S1: Effect sizes for “Prevention” interventions

| **Diseases** | **Procedures** | **Sensitivity** | **Specificity** | **Frequency (per year)** | **Reduction in incidence** | **References** |
| --- | --- | --- | --- | --- | --- | --- |
| **Cervical Cancer** | | | | | | |
| HPV vaccination for types 16 and 18 | |  |  |  | 90%^[[1]](#footnote-1)^ | WHO position paper, Oct 2014 [1]; WHO position paper, Sept 2014 [2] , PRIME [3][ |
| Screening with visual Inspection with acetic acid (VIA) | | 0.66 | 0.77 | 1/3 |  | IARC, 2005 [4], Goldie et al.2001 [5], WHO, 2014 [6] |
| Screening with Papanicolaou (“Pap”) smear | | 0.62 | 0.95 | 1/3 |  | IARC, 2005 [4], Goldie et al., 2001 [5], WHO, 2014 [6] |
| Screening with HPV DNA test | | 0.88 | 0.75 | 1/5 |  | WHO,2014 [6]; IARC, 2005 [4]; Goldie et al., 2001 [5] |
| **Breast Cancer** | | | | | | |
| Screening with Mammography | | 0.76 | 0.93 | 1/2 |  | IARC, 2016 [7], WHO, 2014 [8] |

### Table S2: Effect sizes for “Treatment” interventions

| **Diseases** | **Variables** | | **Stage I** | **Stage II** | **Stage III** | **Stage IV** | **References** |
| --- | --- | --- | --- | --- | --- | --- | --- |
| **Cervical Cancer** | | | | | | | |
| Annual mortality rate | | without treatment | 0.120 | 0.196 | 0.4766 | 1.266 | Goldie et al.,2003 [9], NCCN, 2016 [10], Chuang, 2016 [11] |
|  |  | with treatment | 0.027 | 0.062 | 0.167 | 0.316 |  |
| Impact of treatment (% reduction of mortality) | | | 78% | 68% | 65% | 75% |  |
| Disability weight | | without treatment | 0.3 | 0.3 | 0.3 | 0.57 | Calculated using IHME_GBD_Disability Weight [12] |
|  |  | with treatment | 0.007 | 0.014 | 0.045 | 0.54 |  |
| **Breast Cancer** | | | | | | | |
| Annual mortality rate | | without treatment | 0.14 | 0.18 | 0.23 | 0.5 | Groot et al. 2006 [13]; Zelle et al. 2012 [14], Perez et al. 2014 [15]; Davies et al., 2013, [16]; Feng et al., 2014 [17] |
|  |  | with treatment | 0.006 | 0.039 | 0.093 | 0.27 |  |
| Impact of treatment (% reduction of mortality) | | | 96% | 78% | 60% | 46% |  |
| Disability weight | | without treatment | 0.3 | 0.3 | 0.3 | 0.57 | Calculated using IHME_GBD_Disability Weight [12] |
|  |  | with treatment | 0.008 | 0.008 | 0.029 | 0.54 |  |
| **Colorectal Cancer** | | | | | | | |
| Annual mortality rate | | without treatment | 0.18 | 0.18 | 0.58 | 0.9 | Liu et al., 2014 [18], NCCN, 2017; Frazier et al., 2000 [19]; Wu et al., 2006 [20]; Chadder et al., 2016 [21]; NCIN, 2009 [22]; Seinfeld [23] |
|  |  | with treatment | 0.01 | 0.01 | 0.05 | 0.57 |  |
| Impact of treatment (% reduction of mortality) | | | 94% | 94% | 91% | 37% |  |
| Disability weight | | without treatment | 0.3 | 0.3 | 0.3 | 0.57 | Calculated using IHME_GBD_Disability Weight [12] |
|  |  | with treatment | 0.012 | 0.012 | 0.048 | 0.54 |  |

### Table S3: Stage distribution at diagnosis

| **Diseases** | **Region** | **Stage I** | **Stage II** | **Stage III** | **Stage IV** | **References** |
| --- | --- | --- | --- | --- | --- | --- |
| **Cervical cancer** | | | | | | |
| Eastern sub-Saharan Africa | | 17.12% | 20.42% | 46.33% | 16.14% | Quinn et al.,2006 [24] |
| Southeast Asia | | 19.33% | 36.13% | 39.50% | 5.04% |  |
| **Breast cancer** | | | | | | |
| Eastern sub-Saharan Africa | | 13% | 31% | 39% | 17% | IARC registry data [25]; Sant et al.,2004 [26]; Mandelblatt et al. 2011 [27]; Schwartsmann, 2001 [28]; Chopra, 2001 [29]; Vorobiof et al., 2001 [30]; Groot et al., 2006 [13]; Brinton et al, 2014 [31]; Laurens et al., 2014 [32]; Zelle et al., 2013 [33]; Zelle et al., 2012 [14]; Okonkwo et al., 2008 [34] |
| Southeast Asia | | 17% | 38% | 40% | 5% |  |
| **Colorectal cancer** | | | | | | |
| Eastern sub-Saharan Africa | | 12.30% | 21.90% | 41.90% | 23.90% | Seinfeld [23]; Graham et al., 2012 [35]; Brenner et al. 2016 [36] ; Alsanea et al.,2015 [37]; Zorzi et al., 2015 [38]; Hsu et al., 2015 [39]; IARC registry data [25]; Benitez-Majano et al., 2016 [40] |
| Southeast Asia | | 12.30% | 21.90% | 41.90% | 23.90% |  |

### Table S4: Intervention costing assumptions for Cervical Cancer

| Interventions | Costing components | Cost of Drugs and supplies per person identified/treated (I$ 2010) | Outpatient visits^[[2]](#footnote-2)^ | Inpatient days |
| --- | --- | --- | --- | --- |
| Vaccination against human papillomavirus (2 doses) of 9–13-year-old girls | HPV vaccine price estimated from WHO Prime Tool [3] | 8.52 | 2 | 0 |
| Prevention of cervical cancer by screening women aged 30–49 through visual inspection with acetic acid linked with timely treatment of pre-cancerous lesions | Screening with visual inspection with acetic acid (VIA) performed by trained provider^[[3]](#footnote-3)^ | 2.79 | 1 | 0 |
|  | Same-day treatment of pre-cancerous lesions with cryotherapy for individuals with positive findings on VIA | 10.98 |  |  |
|  | Programme monitoring and evaluation, call and recall mechanism [41] |  |  |  |
| Prevention of cervical cancer by screening women aged 30–49 through Pap smear (cervical cytology) every 3–5 years linked with timely treatment of pre-cancerous lesions | Screening with Papanicolaou (“Pap”) smear performed by trained provider with subsequent review by cytopathologist | 2.64 | 2 | 0 |
|  | Treatment of pre-cancerous lesions (cryotherapy/ loop electrosurgical excision procedure (LEEP)) for individuals with positive findings on colposcopy | 33.47 |  |  |
|  | Programme monitoring and evaluation, call and recall mechanism [41] |  |  |  |
| Prevention of cervical cancer by screening women aged 30–49 through Human papillomavirus test every 5 years linked with timely treatment of pre-cancerous lesions | Screening with HPV DNA test performed by trained provider [42] | 10.34 | 2 | 0 |
|  | Recall for positive HPV test with subsequent visual inspection with acetic acid | 21.03 |  |  |
|  | Same-day treatment of pre-cancerous lesions with cryotherapy for those with positive findings on VIA | 10.98 |  |  |
|  | Programme monitoring and evaluation, call and recall mechanism [41] |  |  |  |
| Treatment of cervical cancer stages I and II with either surgery or radiotherapy +/- chemotherapy | *Diagnosis and staging*: | 18.05 | 7, 10^[[4]](#footnote-4)^ | 6, 2^[[5]](#footnote-5)^ |
|  | Diagnostic evaluation with biopsy, specimen fixative, and staining |  |  |  |
|  | Pre-treatment tests and staging studies when indicated including cross-sectional imaging and ultrasound |  |  |  |
|  | *Treatment* [6], [10], [11]: | 274.93, 1874.65^[[6]](#footnote-6)^ |  |  |
|  | Cone biopsy or simple hysterectomy for microinvasive disease |  |  |  |
|  | Radical hysterectomy for early invasive surgery |  |  |  |
|  | Concurrent chemoradiotherapy with cisplatin and stage IB2 or stage II [43] |  |  |  |
|  | Management of chemotherapy-associated nausea including ondansetron |  |  |  |
|  | *Surveillance with imaging as indicated for 5 years* |  |  |  |
| Basic palliative care for cancer: home-based and hospital care with multi-disciplinary team and access to opiates and essential supportive medicines | Symptom management including amitriptyline, stool softener, morphine (slow release, immediate release), urinary catheter, as needed | 219.75 | 2 | 2 |

### Table S5: Intervention costing assumptions for Breast Cancer

| Interventions | Costing components | Cost of Drugs and supplies per person identified/treated (I$ 2010) | Outpatient visits^[[7]](#footnote-7)^ | Inpatient days |
| --- | --- | --- | --- | --- |
| Treatment of breast cancer stages I and II with surgery +/- systemic therapy | *Diagnosis and staging*: | 116.63 | 8, 10^[[8]](#footnote-8)^ | 2 |
|  | Diagnostic evaluation with biopsy, specimen fixative, and staining |  |  |  |
|  | Biopsy equipment, specimen fixative, and staining |  |  |  |
|  | Pre-treatment tests and staging studies when indicated including x-ray and ultrasound. |  |  |  |
|  | *Treatment*: | 218.01, 464.58^[[9]](#footnote-9)^ |  |  |
|  | Modified radical mastectomy including pre-operative antibiotics, wound drainage kit |  |  |  |
|  | Adjuvant^[[10]](#footnote-10)^ (or neoadjuvant) systemic therapy including doxorubicin, cyclophosphamide, and paclitaxel [44] |  |  |  |
|  | Hormone therapy with tamoxifen^[[11]](#footnote-11)^ |  |  |  |
|  | Management of neutropenia and chemotherapy-associated nausea including filgrastim, ondansetron, and dexamethasone |  |  |  |
|  | *Surveillance with mammogram and clinical exam one visit for 5 years* |  |  |  |
| Screening with mammography (once every 2 years for women aged 50-69 years) linked with timely diagnosis and treatment of breast cancer | *Screening*: |  | 8, 10^[[12]](#footnote-12)^ | 2 |
|  | Screening mammogram | 2.45 |  |  |
|  | Programme monitoring and evaluation, call and recall mechanism [41] |  |  |  |
|  | Management of screen-positive mammograms with subsequent diagnostic studies including mammogram |  |  |  |
|  | *Diagnosis and staging*: | 551.36^[[13]](#footnote-13)^ |  |  |
|  | Biopsy equipment, specimen fixative and staining |  |  |  |
|  | Pre-treatment tests and staging studies when indicated including x-ray and ultrasound |  |  |  |
|  | *Treatment:* | 218.01, 464.58,684.84^[[14]](#footnote-14)^ |  |  |
|  | Modified radical mastectomy including pre-operative antibiotics, wound drainage kit |  |  |  |
|  | Adjuvant systemic therapy including doxorubicin, cyclophosphamide, and paclitaxel^[[15]](#footnote-15)^ |  |  |  |
|  | Hormone therapy with tamoxifen^[[16]](#footnote-16)^ |  |  |  |
|  | *Surveillance with mammogram and clinical exam one visit for 5 years* |  |  |  |
| Basic palliative care for cancer: home-based and hospital care with multi-disciplinary team and access to opiates and essential supportive medicines | Symptom management including amitriptyline, stool softener, morphine (slow release, immediate release), bisphosphonates [45] | 219.75 | 2 | 2 |

### Table S6: Intervention costing assumptions for Colorectal Cancer

| Interventions | Costing components | Cost of Drugs and supplies per person identified/treated (I$ 2010) | Outpatient visits^[[17]](#footnote-17)^ | Inpatient days |
| --- | --- | --- | --- | --- |
| Treatment of colorectal cancer stages I and II with surgery +/- chemotherapy and radiotherapy | *Diagnosis and staging*: | 24.12 | 8, 14^[[18]](#footnote-18)^ | 7 |
|  | Diagnosis with colonoscopy, biopsy, specimen fixative and staining |  |  |  |
|  | Pre-treatment tests and staging studies when indicated including cross-axial imaging |  |  |  |
|  | *Treatment*: | 95.34, 459.88,^[[19]](#footnote-19)^ |  |  |
|  | Colectomy including pre-operative antibiotics |  |  |  |
|  | Adjuvant systemic therapy for colon cancer such as capecitabine and oxaliplatin for select patients with Stage II colon cancer [46] and [44]^[[20]](#footnote-20)^ |  |  |  |
|  | Neoadjuvant systemic therapy for rectal cancer such as capecitabine and radiotherapy for select patients with Stage II rectal cancer [46] and [44]^[[21]](#footnote-21)^ |  |  |  |
|  | Adjuvant chemotherapy with 5-FU, oxaliplatin, and leucovorin for select patients with Stage II rectal cancer [46] and [44]^[[22]](#footnote-22)^ |  |  |  |
|  | Management of complications and toxicities including surgical infection, neutropenia and chemotherapy-associated nausea that includes antibiotics, filgrastim, and ondansetron |  |  |  |
|  | Surveillance includes laboratory test, cross-axial imaging, and endoscopy |  |  |  |
| Basic palliative care for cancer: home-based and hospital care with multi-disciplinary team and access to opiates and essential supportive medicines | Symptom management including amitriptyline, stool softener, morphine (slow release, immediate release), bisphosphonates [45] | 219.75 | 2 | 2 |

*Refer to Table 1 for interventions label

### Table S7: Costs, effects and incremental cost-effectiveness of cervical cancer interventions in Southeast Asia

| **Label*** | **Description of the intervention** | **Pop° coverage (%)** | **Costs per 10 million population (million I$ 2010)** | **HLY per 10 million population (undiscounted)** | **ACER** | | **ICER** |
| --- | --- | --- | --- | --- | --- | --- | --- |
| CVC_C1h | Treatment of cervical cancer stages I and II with either surgery or radiotherapy +/- chemotherapy | 50 | 170 | 171,314 | 993 | Dominated | |
| CVC_C1h | Treatment of cervical cancer stages I and II with either surgery or radiotherapy +/- chemotherapy | 80 | 189 | 335,061 | 565 | Dominated | |
| CVC_C1h | Treatment of cervical cancer stages I and II with either surgery or radiotherapy +/- chemotherapy | 95 | 199 | 445,670 | 447 | Dominated | |
| CVC_C1a | Vaccination against human papillomavirus (2 doses) of 9–13-year-old girls | 50 | 141 | 1,112,285 | 127 | Dominated | |
| CVC_C1a | Vaccination against human papillomavirus (2 doses) of 9–13-year-old girls | 80 | 159 | 1,499,743 | 106 | Dominated | |
| CVC_C1a | Vaccination against human papillomavirus (2 doses) of 9–13-year-old girls | 95 | 169 | 1,630,353 | 103 | Dominated | |
| CVC_C1g | Vaccination against human papillomavirus (2 doses) of 9–13-year-old girls & Prevention of cervical cancer by screening women aged 30–49 through human papillomavirus test every 5 years linked with timely treatment of pre-cancerous lesions | 50 | 363 | 4,284,936 | 85 | Dominated | |
| CVC_C1g | Vaccination against human papillomavirus (2 doses) of 9–13-year-old girls & Prevention of cervical cancer by screening women aged 30–49 through human papillomavirus test every 5 years linked with timely treatment of pre-cancerous lesions | 80 | 487 | 4,927,198 | 99 | Dominated | |
| CVC_C1g | Vaccination against human papillomavirus (2 doses) of 9–13-year-old girls & Prevention of cervical cancer by screening women aged 30–49 through human papillomavirus test every 5 years linked with timely treatment of pre-cancerous lesions | 95 | 549 | 5,109,215 | 108 | Dominated | |
| CVC_C1f | Vaccination against human papillomavirus (2 doses) of 9–13-year-old girls & Prevention of cervical cancer by screening women aged 30–49 through Pap smear (cervical cytology) every 3–5 years linked with timely treatment of pre-cancerous lesions | 50 | 520 | 4,472,666 | 116 | Dominated | |
| CVC_C1f | Vaccination against human papillomavirus (2 doses) of 9–13-year-old girls & Prevention of cervical cancer by screening women aged 30–49 through Pap smear (cervical cytology) every 3–5 years linked with timely treatment of pre-cancerous lesions | 80 | 738 | 5,059,125 | 146 | Dominated | |
| CVC_C1f | Vaccination against human papillomavirus (2 doses) of 9–13-year-old girls & Prevention of cervical cancer by screening women aged 30–49 through Pap smear (cervical cytology) every 3–5 years linked with timely treatment of pre-cancerous lesions | 95 | 847 | 5,222,303 | 162 | Dominated | |
| CVC_C1e | Vaccination against human papillomavirus (2 doses) of 9–13-year-old girls & Prevention of cervical cancer by screening women aged 30–49 through visual inspection with acetic acid linked with timely treatment of pre-cancerous lesions | 50 | 396 | 4,541,842 | 87 | 87 | |
| CVC_C1e | Vaccination against human papillomavirus (2 doses) of 9–13-year-old girls & Prevention of cervical cancer by screening women aged 30–49 through visual inspection with acetic acid linked with timely treatment of pre-cancerous lesions | 80 | 549 | 5,106,391 | 108 | 272 | |
| CVC_C1e | Vaccination against human papillomavirus (2 doses) of 9–13-year-old girls & Prevention of cervical cancer by screening women aged 30–49 through visual inspection with acetic acid linked with timely treatment of pre-cancerous lesions | 95 | 626 | 5,262,580 | 119 | 491 | |
| CVC_C1d | Prevention of cervical cancer by screening women aged 30–49 through human papillomavirus test every 5 years linked with timely treatment of pre-cancerous lesions | 50 | 336 | 3,776,827 | 89 | Dominated | |
| CVC_C1d | Prevention of cervical cancer by screening women aged 30–49 through human papillomavirus test every 5 years linked with timely treatment of pre-cancerous lesions | 80 | 452 | 4,384,869 | 103 | Dominated | |
| CVC_C1d | Prevention of cervical cancer by screening women aged 30–49 through human papillomavirus test every 5 years linked with timely treatment of pre-cancerous lesions | 95 | 510 | 4,565,750 | 112 | Dominated | |
| CVC_C1c | Prevention of cervical cancer by screening women aged 30–49 through Pap smear (cervical cytology) every 3–5 years linked with timely treatment of pre-cancerous lesions | 50 | 493 | 4,002,315 | 123 | Dominated | |
| CVC_C1c | Prevention of cervical cancer by screening women aged 30–49 through Pap smear (cervical cytology) every 3–5 years linked with timely treatment of pre-cancerous lesions | 80 | 703 | 4,554,619 | 154 | Dominated | |
| CVC_C1c | Prevention of cervical cancer by screening women aged 30–49 through Pap smear (cervical cytology) every 3–5 years linked with timely treatment of pre-cancerous lesions | 95 | 807 | 4,714,860 | 171 | Dominated | |
| CVC_C1b | Prevention of cervical cancer by screening women aged 30–49 through visual inspection with acetic acid linked with timely treatment of pre-cancerous lesions | 50 | 368 | 4,085,368 | 90 | Dominated | |
| CVC_C1b | Prevention of cervical cancer by screening women aged 30–49 through visual inspection with acetic acid linked with timely treatment of pre-cancerous lesions | 80 | 513 | 4,615,409 | 111 | Dominated | |
| CVC_C1b | Prevention of cervical cancer by screening women aged 30–49 through visual inspection with acetic acid linked with timely treatment of pre-cancerous lesions | 95 | 585 | 4,767,951 | 123 | Dominated | |
| CVC_C1i | Basic palliative care for cancer: home-based and hospital care with multi-disciplinary team and access to opiates and essential supportive medicines | 50 | 135 | 2,769 | 48,612 | Dominated | |
| CVC_C1i | Basic palliative care for cancer: home-based and hospital care with multi-disciplinary team and access to opiates and essential supportive medicines | 80 | 149 | 4,431 | 33,643 | Dominated | |
| CVC_C1i | Basic palliative care for cancer: home-based and hospital care with multi-disciplinary team and access to opiates and essential supportive medicines | 95 | 156 | 5,262 | 29,704 | Dominated | |

*CVC: Cervical cancer

### Table S8: Costs, effects and incremental cost-effectiveness of breast cancer interventions in Southeast Asia

| **Label*** | **Description of the intervention** | **Pop° coverage (%)** | **Costs per 10 million population ( million I$ 2010)** | **HLY per 10 million population (undiscounted)** | **ACER** | **ICER** |
| --- | --- | --- | --- | --- | --- | --- |
| BRC_C2a | Treatment of breast cancer stages I and II with surgery +/- systemic therapy | 50 | 174 | 335,651 | 517 | Dominated |
| BRC_C2a | Treatment of breast cancer stages I and II with surgery +/- systemic therapy | 80 | 195 | 629,010 | 310 | Dominated |
| BRC_C2a | Treatment of breast cancer stages I and II with surgery +/- systemic therapy | 95 | 206 | 816,200 | 252 | 252 |
| BRC_C2b | Screening with mammography (once every 2 years for women aged 50-69 years) linked with timely diagnosis and treatment of breast cancer | 50 | 618 | 745,528 | 829 | Dominated |
| BRC_C2b | Screening with mammography (once every 2 years for women aged 50-69 years) linked with timely diagnosis and treatment of breast cancer | 80 | 909 | 1,298,852 | 700 | Dominated |
| BRC_C2b | Screening with mammography (once every 2 years for women aged 50-69 years) linked with timely diagnosis and treatment of breast cancer | 95 | 1,056 | 1,627,782 | 649 | 1,048 |
| BRC_C2c | Basic palliative care for cancer: home-based and hospital care with multi-disciplinary team and access to opiates and essential supportive medicines | 50 | 154 | 12,041 | 12,783 | Dominated |
| BRC_C2c | Basic palliative care for cancer: home-based and hospital care with multi-disciplinary team and access to opiates and essential supportive medicines | 80 | 180 | 19,265 | 9,340 | Dominated |
| BRC_C2c | Basic palliative care for cancer: home-based and hospital care with multi-disciplinary team and access to opiates and essential supportive medicines | 95 | 193 | 22,877 | 8,434 | Dominated |

*BRC: Breast cancer

### Table S9: Costs, effects and incremental cost-effectiveness of colorectal cancer interventions in Southeast Asia

| **Label*** | **Description of the intervention** | **Pop° coverage (%)** | **Costs per 10 million population ( million I$ 2010)** | **HLY per 10 million population (undiscounted)** | **ACER** | **ICER** |
| --- | --- | --- | --- | --- | --- | --- |
| CRC_C3a | Treatment of colorectal cancer stages I and II with surgery +/- chemotherapy and radiotherapy | 50 | 174 | 310,289 | 562 | Dominated |
| CRC_C3a | Treatment of colorectal cancer stages I and II with surgery +/- chemotherapy and radiotherapy | 80 | 196 | 633,637 | 310 | Dominated |
| CRC_C3a | Treatment of colorectal cancer stages I and II with surgery +/- chemotherapy and radiotherapy | 95 | 207 | 870,417 | 238 | 238 |
| CRC_C3b | Basic palliative care for cancer: home-based and hospital care with multi-disciplinary team and access to opiates and essential supportive medicines | 50 | 135 | 3,128 | 43,307 | Dominated |
| CRC_C3b | Basic palliative care for cancer: home-based and hospital care with multi-disciplinary team and access to opiates and essential supportive medicines | 80 | 150 | 5,006 | 30,058 | Dominated |
| CRC_C3b | Basic palliative care for cancer: home-based and hospital care with multi-disciplinary team and access to opiates and essential supportive medicines | 95 | 158 | 5,944 | 26,571 | Dominated |

*CRC: Colorectal cancer

### Table S10: Costs, effects and incremental cost-effectiveness of cervical cancer interventions in Eastern sub-Saharan Africa

| **Label*** | **Description of the intervention** | **Pop° coverage (%)** | **Costs per 10 million population  ( million I$ 2010)** | **HLY per 10 million population (undiscounted)** | **ACER** | **ICER** |
| --- | --- | --- | --- | --- | --- | --- |
| CVC_C1h | Treatment of cervical cancer stages I and II with either surgery or radiotherapy +/- chemotherapy | 50 | 164 | 918,353 | 179 | Dominated |
| CVC_C1h | Treatment of cervical cancer stages I and II with either surgery or radiotherapy +/- chemotherapy | 80 | 211 | 1,777,983 | 119 | Dominated |
| CVC_C1h | Treatment of cervical cancer stages I and II with either surgery or radiotherapy +/- chemotherapy | 95 | 235 | 2,355,450 | 100 | Dominated |
| CVC_C1a | Vaccination against human papillomavirus (2 doses) of 9–13-year-old girls | 50 | 146 | 5,215,136 | 28 | 28 |
| CVC_C1a | Vaccination against human papillomavirus (2 doses) of 9–13-year-old girls | 80 | 190 | 6,773,262 | 28 | 28 |
| CVC_C1a | Vaccination against human papillomavirus (2 doses) of 9–13-year-old girls | 95 | 213 | 7,297,912 | 29 | Dominated |
| CVC_C1g | Vaccination against human papillomavirus (2 doses) of 9–13-year-old girls & Prevention of cervical cancer by screening women aged 30–49 through human papillomavirus test every 5 years linked with timely treatment of pre-cancerous lesions | 50 | 697 | 24,649,274 | 28 | Dominated |
| CVC_C1g | Vaccination against human papillomavirus (2 doses) of 9–13-year-old girls & Prevention of cervical cancer by screening women aged 30–49 through human papillomavirus test every 5 years linked with timely treatment of pre-cancerous lesions | 80 | 1,043 | 29,121,530 | 36 | Dominated |
| CVC_C1g | Vaccination against human papillomavirus (2 doses) of 9–13-year-old girls & Prevention of cervical cancer by screening women aged 30–49 through human papillomavirus test every 5 years linked with timely treatment of pre-cancerous lesions | 95 | 1,213 | 30,413,350 | 40 | Dominated |
| CVC_C1f | Vaccination against human papillomavirus (2 doses) of 9–13-year-old girls & Prevention of cervical cancer by screening women aged 30–49 through Pap smear (cervical cytology) every 3–5 years linked with timely treatment of pre-cancerous lesions | 50 | 1,071 | 25,894,136 | 41 | Dominated |
| CVC_C1f | Vaccination against human papillomavirus (2 doses) of 9–13-year-old girls & Prevention of cervical cancer by screening women aged 30–49 through Pap smear (cervical cytology) every 3–5 years linked with timely treatment of pre-cancerous lesions | 80 | 1,639 | 30,073,810 | 55 | Dominated |
| CVC_C1f | Vaccination against human papillomavirus (2 doses) of 9–13-year-old girls & Prevention of cervical cancer by screening women aged 30–49 through Pap smear (cervical cytology) every 3–5 years linked with timely treatment of pre-cancerous lesions | 95 | 1,920 | 31,251,433 | 61 | Dominated |
| CVC_C1e | Vaccination against human papillomavirus (2 doses) of 9–13-year-old girls & Prevention of cervical cancer by screening women aged 30–49 through visual inspection with acetic acid linked with timely treatment of pre-cancerous lesions | 50 | 764 | 26,362,292 | 29 | Dominated |
| CVC_C1e | Vaccination against human papillomavirus (2 doses) of 9–13-year-old girls & Prevention of cervical cancer by screening women aged 30–49 through visual inspection with acetic acid linked with timely treatment of pre-cancerous lesions | 80 | 1,163 | 30,421,065 | 38 | 41 |
| CVC_C1e | Vaccination against human papillomavirus (2 doses) of 9–13-year-old girls & Prevention of cervical cancer by screening women aged 30–49 through visual inspection with acetic acid linked with timely treatment of pre-cancerous lesions | 95 | 1,362 | 31,554,286 | 43 | 175 |
| CVC_C1d | Prevention of cervical cancer by screening women aged 30–49 through human papillomavirus test every 5 years linked with timely treatment of pre-cancerous lesions | 50 | 621 | 21,058,982 | 29 | Dominated |
| CVC_C1d | Prevention of cervical cancer by screening women aged 30–49 through human papillomavirus test every 5 years linked with timely treatment of pre-cancerous lesions | 80 | 919 | 25,096,943 | 37 | Dominated |
| CVC_C1d | Prevention of cervical cancer by screening women aged 30–49 through human papillomavirus test every 5 years linked with timely treatment of pre-cancerous lesions | 95 | 1,064 | 26,370,394 | 40 | Dominated |
| CVC_C1c | Prevention of cervical cancer by screening women aged 30–49 through Pap smear (cervical cytology) every 3–5 years linked with timely treatment of pre-cancerous lesions | 50 | 995 | 22,516,816 | 44 | Dominated |
| CVC_C1c | Prevention of cervical cancer by screening women aged 30–49 through Pap smear (cervical cytology) every 3–5 years linked with timely treatment of pre-cancerous lesions | 80 | 1,514 | 26,290,979 | 58 | Dominated |
| CVC_C1c | Prevention of cervical cancer by screening women aged 30–49 through Pap smear (cervical cytology) every 3–5 years linked with timely treatment of pre-cancerous lesions | 95 | 1,769 | 27,447,414 | 64 | Dominated |
| CVC_C1b | Prevention of cervical cancer by screening women aged 30–49 through visual inspection with acetic acid linked with timely treatment of pre-cancerous lesions | 50 | 687 | 23,064,846 | 30 | Dominated |
| CVC_C1b | Prevention of cervical cancer by screening women aged 30–49 through visual inspection with acetic acid linked with timely treatment of pre-cancerous lesions | 80 | 1,038 | 26,726,375 | 39 | Dominated |
| CVC_C1b | Prevention of cervical cancer by screening women aged 30–49 through visual inspection with acetic acid linked with timely treatment of pre-cancerous lesions | 95 | 1,210 | 27,836,622 | 43 | Dominated |
| CVC_C1i | Basic palliative care for cancer: home-based and hospital care with multi-disciplinary team and access to opiates and essential supportive medicines | 50 | 119 | 25,520 | 4,654 | Dominated |
| CVC_C1i | Basic palliative care for cancer: home-based and hospital care with multi-disciplinary team and access to opiates and essential supportive medicines | 80 | 147 | 40,832 | 3,595 | Dominated |
| CVC_C1i | Basic palliative care for cancer: home-based and hospital care with multi-disciplinary team and access to opiates and essential supportive medicines | 95 | 161 | 48,488 | 3,316 | Dominated |

*CVC: Cervical cancer

### Table S11: Costs, effects and incremental cost-effectiveness of breast cancer interventions in Eastern sub-Saharan Africa

| **Label*** | **Description of the intervention** | **Pop° coverage (%)** | **Costs per 10 million population ( million I$ 2010)** | **HLY per 10 million population (undiscounted)** | **ACER** | **ICER** |
| --- | --- | --- | --- | --- | --- | --- |
| BRC_C2a | Treatment of breast cancer stages I and II with surgery +/- systemic therapy | 50 | 123 | 584,274 | 211 | Dominated |
| BRC_C2a | Treatment of breast cancer stages I and II with surgery +/- systemic therapy | 80 | 146 | 1,080,913 | 135 | Dominated |
| BRC_C2a | Treatment of breast cancer stages I and II with surgery +/- systemic therapy | 95 | 157 | 1,389,662 | 113 | 113 |
| BRC_C2b | Screening with mammography (once every 2 years for women aged 50-69 years) linked with timely diagnosis and treatment of breast cancer | 50 | 721 | 1,237,705 | 582 | Dominated |
| BRC_C2b | Screening with mammography (once every 2 years for women aged 50-69 years) linked with timely diagnosis and treatment of breast cancer | 80 | 1,110 | 2,159,801 | 514 | Dominated |
| BRC_C2b | Screening with mammography (once every 2 years for women aged 50-69 years) linked with timely diagnosis and treatment of breast cancer | 95 | 1,307 | 2,697,617 | 485 | 879 |
| BRC_C2c | Basic palliative care for cancer: home-based and hospital care with multi-disciplinary team and access to opiates and essential supportive medicines | 50 | 124 | 29,868 | 4,152 | Dominated |
| BRC_C2c | Basic palliative care for cancer: home-based and hospital care with multi-disciplinary team and access to opiates and essential supportive medicines | 80 | 155 | 47,789 | 3,247 | Dominated |
| BRC_C2c | Basic palliative care for cancer: home-based and hospital care with multi-disciplinary team and access to opiates and essential supportive medicines | 95 | 171 | 56,749 | 3,009 | Dominated |

*BRC: Breast cancer

### Table S12: Costs, effects and incremental cost-effectiveness of colorectal cancer interventions in Eastern sub-Saharan Africa

| **Label*** | **Description of the intervention** | **Pop° coverage (%)** | **Costs per 10 million population ( million I$ 2010)** | **HLY per 10 million population (undiscounted)** | **ACER** | **ICER** |
| --- | --- | --- | --- | --- | --- | --- |
| CRC_C3a | Treatment of colorectal cancer stages I and II with surgery +/- chemotherapy and radiotherapy | 50 | 112 | 233,095 | 480 | Dominated |
| CRC_C3a | Treatment of colorectal cancer stages I and II with surgery +/- chemotherapy and radiotherapy | 80 | 128 | 464,692 | 275 | Dominated |
| CRC_C3a | Treatment of colorectal cancer stages I and II with surgery +/- chemotherapy and radiotherapy | 95 | 136 | 626,379 | 217 | 217 |
| CRC_C3b | Basic palliative care for cancer: home-based and hospital care with multi-disciplinary team and access to opiates and essential supportive medicines | 50 | 93 | 2,949 | 31,699 | Dominated |
| CRC_C3b | Basic palliative care for cancer: home-based and hospital care with multi-disciplinary team and access to opiates and essential supportive medicines | 80 | 106 | 4,718 | 22,530 | Dominated |
| CRC_C3b | Basic palliative care for cancer: home-based and hospital care with multi-disciplinary team and access to opiates and essential supportive medicines | 95 | 113 | 5,602 | 20,117 | Dominated |

*CRC: Colorectal cancer

## References

[1] World Health Organization. Human papillomavirus vaccines: WHO position paper. Weekly epidemiological record. 2014; 89(43): 465-92.

[2] World Health Organization. Efficacy of HPV vaccination in adolescent girls: WHO position paper. 2014. <http://www.who.int/immunization/position_papers/hpv_grad_efficacy_young_females.pdf>. Accessed 2016.

[3] Jit M, Brisson M, Portnoy A, Hutubessy R. Cost-effectiveness of female human papillomavirus vaccination in 179 countries: a PRIME modelling study. The Lancet Global Health. 2014; 2(7): 406-14.

[4] International Agency for Research on Cancer. IARC Handbooks of Cancer Prevention: Cervix Cancer Screening. Vol. 10; 2005.

[5] Goldie S, Kuhn L, Denny L, Pollack A, Wright T. Policy Analysis of Cervical Cancer Screening Strategies in Low-Resource Settings: Clinical Benefits and Cost-effectiveness. JAMA. 2001; 285(24): 3107-115.

[6] World Health Organization.Comprehensive cervical cancer control: a guide to essential practice. 2nd ed; 2014.

[7] International Agency for Research on Cancer. IARC Handbook of Cancer Prevention: Breast Cancer Screening. Vol. 15; 2016.

[8] World Health Organization. WHO position paper on mammography screening. Geneva: World Health Organization; 2014.

[9] Goldie S, Grima D, Kohli M, Wright T, Weinstein M, Franco E. A comprehensive natural history model of HPV infection and cervical cancer to estimate the clinical impact of prophylactic HPV-16/18 Vaccine. International Journal of Cancer. 2003; 106(6): 896-904.

[10] National Comprehensive Cancer Network. Cervical Cancer: Guidelines for Treatment. 2016. https://www.nccn.org/professionals/physician_gls/f_guidelines.asp#cervical. Accessed 1 June 2016.

[11] Chuang L, Temin S, Berek J. Management and care of women with invasive cervical cancer: american society of clinical oncology resource-stratified clinical practice guideline summary. Journal of Global Oncology. 2016; 12(7): 693-6.

[12] Murray C, Vos T, Lozano R, Naghavi M, Flaxman A, Michaud C, et al. Disability-adjusted life years (DALYs) for 291 diseases and injuries in 21 regions, 1999-2010: a systematic analysis for the Global Burden of Disease Study 2010. The Lancet. 2012; 380(9859): 2197-223.

[13] Groot MT, Baltussen R, Uyl-de Groot CA, Anderson BO, Hortobágyi GN. Costs and Health Effects of Breast Cancer Interventions in Epidemiologically Different Regions of Africa, North America, and Asia. The Breast Journal. 2006; 12(s1): S81-S90.

[14] Zelle SG, Nyarko KM, Bosu WK, Aikins M, Niens LM, Lauer JA, Sepulveda CR, Hontelez JAC, Baltussen R. Costs, effects and cost-effectiveness of breast cancer control in Ghana. Tropical Medicine and International Health. 2012; 17(8): 1031-43.

[15] Perez E, Romond E, Suman V, Jeong J, Sledge G, . Geyer CJ, Martino S, Rastogi P, Gralow J, Swain S, . Winer E, Colon-Otero G, Davidson N, Mamounas E, Zujewsk J , Wolmark N. Trastuzumab plus adjuvant chemotherapy for human epidermal growth factor receptor 2-positive breast cancer: planned joint analysis of overall survival from NSABP B-31 and NCCTG N9831. Journal of Clinical Oncology. 2014; 32(33): 3744-52.

[16] Davies C , Pan H, Godwin J, Gray R, Arriagada R, et al. Long-term effects of continuing adjuvant tamoxifen to 10 years versus stopping at 5 years after diagnosis of oestrogen receptor-positive breast cancer: ATLAS, a randomised trial. The Lancet. 2013; 381(9869): 805-16.

[17] Wen F, Yao K, Du ZD, He XF, Zhang PF, Tang RL, Li Q. Cost-effectiveness analysis of colon cancer treatments from MOSIAC and No. 16968 trials. World Journal Gastroenterology. 2014; 20(47): 17976-84.

[18] Liu CY, Chen WTL, P.-T. Kun, C.-F. Chiu, Y.-H. Wang, S.-H. Shieh and W.-C. Tsai, “Characteristics, survival, and related factors of newly diagnosed colorectal cancer patients refusing cancer treatments under a universal health insurance program,” BMC Cancer, vol. 14, no. 446, 2014.

[19] Frazier AL, Colditz GA, Fuchs CS, et al. Cost-effectiveness of Screening for Colorectal Cancer in the General Population. JAMA. 2000; 284(15): 1954-61.

[20] Wu GHM, Wang YM, Yen AMF, Wong JM, Lai HC, Warwick J, Chen THH. Cost-effectiveness analysis of colorectal cancer screening with stool DNA testing in intermediate-incidence countries. BMC Cancer. 2006; 6:136.

[21] Chadder J, Dewar R, Shack L, Nishri D, Niu J, Lockwood G. A first look at relative survival by stage for colorectal and lung cancers in Canada. Current Oncology. 2016; 23(2): 119-24.

[22] National Cancer Intelligence Network. Colorectal Cancer Survival by Stage - NCIN Data Briefing. In: National Cancer Registration and Analysis Service. 2009. <http://www.ncin.org.uk/publications/data_briefings/colorectal_cancer_survival_by_stage>. Accessed 2016.

[23] Seinfeld J. Cost-benefit analysis of cancer care and control: The case of cervical, colorectal and breast cancer in low and middle income countries. https://pdfs.semanticscholar.org/af32/64a332f565cb6581d19af76ca214a081855f.pdf. Accessed 2016.

[24] Quinn MA, Benedet JL, Odicino F, Maisonneuve P, Beller U, Creasman WT, Heintz AP, Ngan HY, Pecorelli S. Carcinoma of the cervix uteri. FIGO 26th Annual report on the results of treatment in gynecological cancer. International Journal of Gynecology and Obstetrics. 2006; 95(s1): S43-103.

[25] IARC Registry Data. International Agency for Research on Cancer. http://survcan.iarc.fr/survivalstats.php?country=chiangmai&table=Table5b&soumission=subm. Accessed 2016.

[26] Sant M, Allemani C, Berrino F, Coleman M, Aareleid T, et al. Breast Carcinoma Survival in Europe and the United States. Cancer. 2004; 100(4): 715-22.

[27] Mandelblatt JS, Cronin KA, Berry DA, Chang Y, de Koning HJ, Lee SJ, Plevritis SK, Schechter CB, Stout NK, van Ravesteyn NT, Zelen M, Feuer EJ. Modeling the impact of population screening on breast cancer mortality in the United States. Breast. 2011; 20(s3): S75:81.

[28] Schwartsmann G. Breast cancer in South America: challenges to improve early detection and medical management of a public health problem. Journal of Clinical Oncology. 2001; 19(18 Suppl):118S-124S.

[29] Chopra R. The Indian Scene. Journal of Clinical Oncology. 2001; 19(18 Suppl):106S-111S.

[30] Vorobiof D, Sitas F, Vorobiof G. Breast Cancer Incidence in South Africa. Journal of Clinical Oncology. 2001;19(18 Suppl): 125S-127S.

[31] Brinton LA, Figueroa JD, Awuah B, Yarney J, Wiafe S, Wood SN, Ansong D, Nyarko K, Wiafe-Addai B, Clegg-Lamptey JN. Breast Cancer in Sub Saharan Africa: Opportunities for prevention. Breast Cancer Research and Treatment. 2014; 144(3): 467-78.

[32] Nïens LM, Zelle SG, Gutiérrez-Delgado C, Peña GR, Balarezo BRH, Steller ER, Rutten FFH. Cost-effectiveness of breast cancer control strategies in Central America: the cases of Costa Rica and Mexico. PLOS One. 2014; [doi.org/10.1371/journal.pone.0095836](https://doi.org/10.1371/journal.pone.0095836).

[33] Zelle SG, Vidaurre T, Abugattas JE, Manrique JE, Sarria G, Jeronimo J, Seinfeld JN, Lauer JA, Sepulveda CR, Venegas D, Baltussen R. Cost-Effectiveness Analysis Of Breast Cancer Control Interventions In Peru. PLOS One. 2013; doi.org/10.1371/journal.pone.0082575.

[34] Okonkwo Q, Draisma G, der Kinderen A, Brown M, de Koning H. Breast cancer screening policies in developing countries: a cost-effectiveness analysis for India. Journal of the National Cancer Institute. 2008; 100(18):1290-1300.

[35] Graham A, Adeloye D, Grant L, Theodoratou E, Campbell H. Estimating the incidence of colorectal cancer in Sub–Saharan Africa: A systematic analysis. Journal of Global Health. 2012; 2(2):020404.

[36] Brenner H, Jansen L, Ulrich A, Chang-Claude J, Hoffmeister M. Survival of patients with symptom- and screening-detected colorectal cancer. Oncotarget. 2016; 7(28): 44695-704.

[37] Alsanea N, Abduljabbar AS, Alhomoud S, Ashari LH, Hibbert D, Bazarbashi S. Colorectal cancer in Saudi Arabia: incidence, survival, demographics and implications for national policies. Annals of Saudi Medicine.2015; 35(3): 196-202.

[38] Zorzi M, Mangone L, Anghinon E, Baracco S, Borciani E, Caldarella A, Falcini F, Fanetti AC, Ferretti S, . Rossi PG, Michiara M, Randi G, Stracci F, Vicentini M, Zucchetto A, Zappa M, IMPATTO COLONRETTO working group. Characteristics of the colorectal cancers diagnosed in the early 2000s in Italy. Figures from the IMPATTO study on colorectal cancer screening. Epidemiol Prev. 2015; 39(3) Suppl 1:1-125.

[39] Hsu YH, Kung PT, Wang YH, Chang YM, Tsai WC. A comparison of the stages at which cancer is diagnosed in physicians and in the general population in Taiwan. CMAJ. 2015;187(13): E412–8.

[40] Benitez-Majano S, Fowler H, Maringe C, Di Girolamo C, Rachet B. Deriving stage at diagnosis from multiple population-based sources: colorectal and lung cancer in England. British Journal of Cancer. 2016; 115(3): 391-400.

[41] European Science Advisory Network for Health. Determinants for a successful implementation of population-based cancer screening programmes (EuSANH-ISA, 229716). European Science Advisory Network for Health; 2011.

[42] Levin C, Sellors J, Shi J, Ma L, Qiao Y, Ortendahl J, O'Shea M, Goldie S. Cost-effectiveness analysis of cervical cancer prevention based on a rapid human papillomavirus screening test in a high-risk region of China. International Journal of Cancer. 2010; 127(6): 1404-11.

[43] Atun R, Jaffray D, Barton M, Bray F, Baumann M, Vikram B, Hanna T, Knaul F, Lievens Y, Lui T, Milosevic M, O'Sullivan B, Rodin D, Rosenblatt E, Van Dyk J, Yap M, Zubizarreta E, Gospodarowicz M. Expanding global access to radiotherapy. The Lancet Oncology. 2015; 16(10):1153-86.

[44] World Health Organization. WHO Model List of Essential Medicines. 19^th^ edition .Geneva: World Health Organization; 2015.

[45] World Health Organization. Planning and implementing palliative care services: a guide for programme managers. Geneva: World Health Organization; 2016.

[46] National Comprehensive Cancer Network. Colon Cancer. Clinical Practice Guidelines in Oncology (NCCN Guidelines).Version 2. 2017. http://www.nccn.org . Accessed 1 July 2016.

[47] Clifford G, Gallus S, Herrero R, Munoz N, Snijders P, Vaccarella S, et al. Worldwide distribution of human papillomavirus types in cytologically normal women in the International Agency for Research on Cancer HPV prevalence surveys: a pooled analysis. The Lancet.2005; 366(9490):991-8.

[48] Bruni L , Diaz M, Castellsagué X, Ferrer E, Bosch FX, de Sanjosé S. Cervical human papillomavirus prevalence in 5 continents: meta-analysis of 1 million women with normal cytological findings. The Journal of Infectious Diseases. 2010;202(12):1789-99.

[49] Smith J, Lindsay L, Hoots B, Keys J, Franceschi S, Winer R, et al. Human papillomavirus type distribution in invasive cervical cancer and high-grade cervical lesions: a meta-analysis update. International Journal of Cancer. 2007;121(3): 621-32.

1. 90% effectiveness for types 16 and 18 as used in WHO PRIME tool [3]. Estimated Incidence of HPV types 16 and 18 taken from [47], [48], [49] [↑](#footnote-ref-1)
2. Costing includes health workforce time and outpatient facility visit. [↑](#footnote-ref-2)
3. Referral for subsequent colposcopy and/or biopsy for suspicious lesions [↑](#footnote-ref-3)
4. 7 visits for stage I, 10 visits for stage II [↑](#footnote-ref-4)
5. 6 days for stage I, 2 days for stage II [↑](#footnote-ref-5)
6. 274.93 I$ for stage I, 1874.65 I$ for stage II [↑](#footnote-ref-6)
7. Costing includes health workforce time and outpatient facility visit. [↑](#footnote-ref-7)
8. 8 days for stage I, 10 days for stage II [↑](#footnote-ref-8)
9. 218.01 for Stage I, 464.58 for Stage II [↑](#footnote-ref-9)
10. Given to 5% of stage I patients and 30% of stage II patients [↑](#footnote-ref-10)
11. Needed for patients with hormone receptor positive cancers (estimated at 40%) [↑](#footnote-ref-11)
12. 8 days for stage I, 10 days for stage II [↑](#footnote-ref-12)
13. Diagnostic costs include false positive findings requiring subsequent diagnostic mammography with or without biopsy [↑](#footnote-ref-13)
14. 218.01 for Stage I, 464.58 for Stage II, 684.84 for Stage III [↑](#footnote-ref-14)
15. Adjuvant therapy is given to 5% of stage I patients, 30% of stage II patients, and 60% of stage III [↑](#footnote-ref-15)
16. Needed for patients with hormone receptor positive cancers (estimated at 40%) [↑](#footnote-ref-16)
17. Costing includes health workforce time and outpatient facility visit. [↑](#footnote-ref-17)
18. 8 for stage I, 14 for stage II [↑](#footnote-ref-18)
19. 95.34 for Stage I, 459.88 for Stage II [↑](#footnote-ref-19)
20. Estimated at 10% of stage II colon cancer patients require systemic therapy. [↑](#footnote-ref-20)
21. Estimated at 50% of stage II rectal cancer patients require neoadjuvant systemic therapy [↑](#footnote-ref-21)
22. Estimated at 10% of stage II rectal cancer patients require adjuvant systemic therapy. [↑](#footnote-ref-22)
